# Accessory proteins for heterotrimeric G-proteins in the kidney

**DOI:** 10.3389/fphys.2015.00219

**Published:** 2015-08-07

**Authors:** Frank Park

**Affiliations:** Department of Pharmaceutical Sciences, College of Pharmacy, University of Tennessee Health Science CenterMemphis, TN, USA

**Keywords:** G-proteins, accessory proteins, kidney, signal transduction, polycystic kidney disease, acute kidney injury

## Abstract

Heterotrimeric G-proteins play a fundamentally important role in regulating signal transduction pathways in the kidney. Accessory proteins are being identified as direct binding partners for heterotrimeric G-protein α or βγ subunits to promote more diverse mechanisms by which G-protein signaling is controlled. In some instances, accessory proteins can modulate the signaling magnitude, localization, and duration following the activation of cell membrane-associated receptors. Alternatively, accessory proteins complexed with their G-protein α or βγ subunits can promote non-canonical models of signaling activity within the cell. In this review, we will highlight the expression profile, localization and functional importance of these newly identified accessory proteins to control the function of select G-protein subunits under normal and various disease conditions observed in the kidney.

## Introduction

The kidney plays a fundamentally important role in controlling a wide array of physiological processes, including fluid and electrolyte balance, hormone production, acid-base balance, and blood pressure regulation (Alpern et al., 2013). In many of these biological functions, heterotrimeric G-proteins play a crucial role through the stimulation of cell surface G-protein coupled receptors (GPCRs) to activate diverse signaling networks depending upon their association with distinct G-protein subunits (Wettschureck and Offermanns, 2005; Insel et al., 2007). To date, GPCRs are a family of seven-transmembrane spanning integral membrane proteins with ~800 different members (Hollinger and Hepler, 2002), and are a common target for drug development. Canonical signaling from GPCR activation was initially described as a ligand dependent mechanism that used heterotrimeric G-proteins as an intermediary controller in the activation of intracellular signaling cascades. In this mode of action, the GPCR functions as a guanine exchange factor (GEF) to facilitate the replacement of GDP with GTP on the Gα subunit, and leads to a conformational change in the heterotrimeric G-protein structure to promote effector system activation by Gα-GTP and unbound Gβγ dimers. The intrinsic GTPase activity of the Gα subunit controls the rate by which GTP is hydrolyzed to GDP resulting in re-association with Gβγ to inactivate heterotrimeric G-protein function.

Over the past two decades, there is increasing evidence that the classic GPCR activation of heterotrimeric G-proteins is more complex than original anticipated. The activation status of the heterotrimeric G-protein can depend on a number of factors, including phosphorylation by kinases (Tobin, 2008), adaptor proteins (Ritter and Hall, 2009; Magalhaes et al., 2012), and more recently, the identification of accessory proteins that can selectively bind to individual G-protein subunits to regulate their activation status independent of GPCR input (Hollinger and Hepler, 2002; Blumer et al., 2005, 2007). It is becoming increasingly more evident that the complexity of signaling output within the kidney is dependent upon the repertoire of GPCR, G-protein subunits, and accessory proteins located within an individual tissue and possibly each cell type.

The regulation by accessory proteins on heterotrimeric G-proteins has shown little fanfare in the kidney. However, there are an increasing number of studies that have demonstrated the induction of previously unstudied accessory proteins in the glomerulus, blood vessels or tubular epithelial cells leading to an exaggerated response to a biological insult. In the following sections of this review and summarized in Table 1, we will categorize the accessory proteins in terms of its mode of action, and where available, discuss their biological role in normal and various disease states of the kidney, including cystic disease, acute kidney injury, fibrosis, blood pressure regulation, and cancer.

**Table 1:** **Accessory proteins for heterotrimeric G-proteins in the kidney**.

**Functional role**	**Accessory protein**	**G-protein subunit**	**Function**	**References**
**I. NORMAL PHYSIOLOGY**				
Receptor desensitization	GRK2/3	Gα_q/11_, Gβγ	Promotes redistribution to plasma membrane to inactivate GPCR signaling	Kamal et al., 2012; Sato et al., 2015
Cytoskeleton	Tubulin	Gα_q_, Gα_*i*1_, G_s_	Gα-GTP promotes microtubule	Roychowdhury and Rasenick, 1994; Yan et al., 1996; Roychowdhury et al., 1999; Schappi et al., 2014
		Gβγ	Disassembly; Gβγ promotes microtubule polymerization	
	Radixin	Gα_13_-GTP	Promotes conformational change to bind F-actin	Vaiskunaite et al., 2000
Second messenger system	RGS2	Gα_*s*_, Gα_q_	Partially inhibits water reabsorption by direct interaction between RGS2 with Gα_s_ and adenylyl cyclase isoforms at the plasma membrane	Roy et al., 2003, 2006; Zuber et al., 2007
Ion transporters	AGS11/TFE3	Gα_16_	Regulates Npt2 expression	Miyamoto and Itho, 2001
	EBP50/NHERF1	Gα_q_	Gα_q_ inhibits PLC-β1 signaling by binding to the same PDZ domains as PLC; dependency on G-protein binding to alter sodium and phosphate transport remains to be determined	Rochdi et al., 2002; Morales et al., 2004
Glomerular function	HSP90	Gα_12_, Gβγ	Regulate GFR through NO-dependent mechanism; role of G-proteins remains to be determined	Inanobe et al., 1994; Vaiskunaite et al., 2001; Ramirez et al., 2008
**II. PATHOPHYSIOLOGY/KIDNEY DISEASE**				
Cystic kidney disease	RGS7	Gβ_5_	Interacts with C-terminal tail of PC-1; function unknown	Kim et al., 1999
	AGS3/GPSM1	Gα_i/o_	Attenuates cystic disease progression; activates heteromeric PC1/PC2 ion channel	Kwon et al., 2012; Nadella et al., 2010
	AGS5/LGN	Gα_i/o_	Loss of AGS5/LGN promotes abnormal cyst formation in MDCK cells	Zheng et al., 2010; Xiao et al., 2012
	AGS11/TFE3	Gα_16_	Associated with appearance of cysts in BHD; mechanism not known	Luijten et al., 2013
	HSP90	Gα_12_, Gβγ	Accelerates cystic disease; role of G-proteins not known	Seeger-Nukpezah et al., 2013
Acute kidney injury	RGS4	Gα_i_	Lack of RGS4 exacerbates the reduction in total blood flow after IRI; RGS4 competes for activated Gα_q_ by ANGII to prevent secretion of RANTES	Siedlecki et al., 2011; Pang et al., 2015
	AGS1/Dexras1/RasD1		No known function	Rusai et al., 2013; Zhang et al., 2013
	AGS3/GPSM1	Gα_i/o_	Promotes tubular epithelial cell repair following IRI	Regner et al., 2011; Lenarczyk et al., 2015
	RACK1	Gβγ	Induction of proximal tubular RACK1 expression following IRI; functional role is not known	Padanilam and Hammerman, 1997
	HSP90	Gα_12_	HSP90 induction in proximal tubules after IRI; blockade of HSP90 activity reduces epithelial cells damage after IRI, but due to compensatory induction of other HSPs; over-expression of HSP90 restores coupling with eNOS after IRI to limit the extent of epithelial cell damage after IRI; role of G-protein interaction remains to be determined	Vaiskunaite et al., 2001
Glomerular injury/disease	RGS2	Gα_q_	RGS2 negatively regulates urotensin II-dependent calcium increase and contraction	Adebiyi, 2014
	GIV/Girdin	Gα_i/o_	Protects glomerulus from injury by activating PI3K/Akt pathway	Wang et al., 2015
	Rap1GAP	Gα_i/o_	Increased expression in podocytes from injury glomeruli promoted cellular detachment by inhibition activation of β1 integrin	Potla et al., 2014
Renal cancer	AGS11/TFE3	Gα_16_	Associated with nuclear localization of hybrid TFE3-fusion proteins in translocation renal cell carcinoma	Meloni et al., 1993; Weterman et al., 1996a,b, 2000, 2001; Kuiper et al., 2003; Mathur and Samuels, 2007
	Rap1GAP	Gα_i/o_	Loss of Rap1GAP promotes abnormal cell migration and invasion	Kim et al., 2012
	RGS5	Gα_i/o_	Selectively expressed in the blood vessel in renal cell carcinoma; may promote angiogenesis	Furuya et al., 2004
Fibrosis	RGS2	Gα_q_	Slows onset of renal fibrosis by blocking the AT1R activated pro-fibrogenic and inflammatory systems	Jang et al., 2014
Vascular hyperactivity and hypertension	RGS2	Gα_q_	Loss of renal RGS2 produces mild hypertension	Gurley et al., 2010
	RACK1	Gβγ	Hyperactive PLC/PKC increases vascular cell proliferation in in hypertensive rats *in vitro*	Cheng et al., 2011

## Types of accessory proteins in the normal kidney

**Figure 1:**
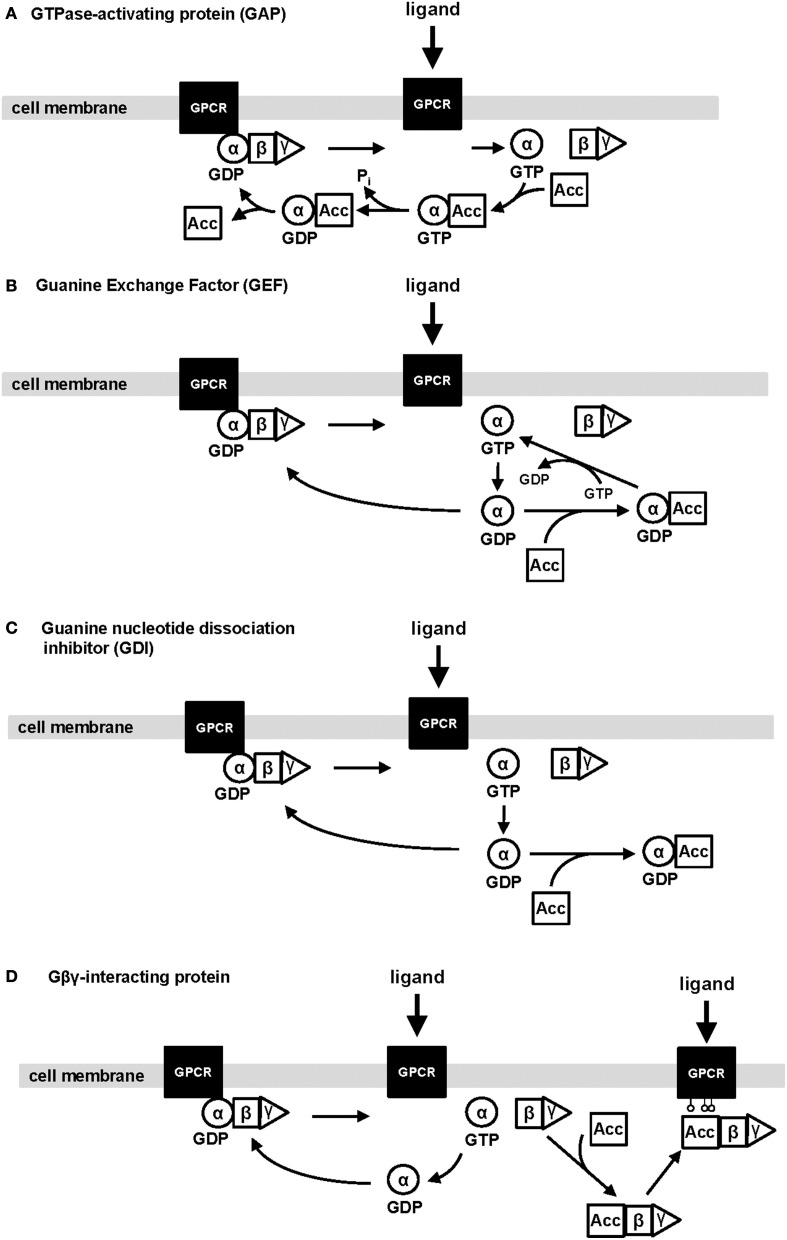
**Schematic illustrating the four types of regulation exhibited by accessory proteins on heterotrimeric G-protein subunits**. The four main types of regulation mediated by accessory proteins are: (1) GTPase-activating proteins; (2) Guanine exchange factors; (3) Guanine nucleotide dissociation inhibitors; and (4) Gβγ-interacting proteins. Ligand activation promotes a conformational change in the heterotrimeric G-protein associated with the GPCR, which facilitates the switch from GDP to GTP-bound Gα subunits. Subsequently, this leads to the activation of downstream effector systems by Gα-GTP and unbound Gβγ. The Gα subunit has intrinsic GTPase activity to inactivate the signaling output and ultimately, reassociate with its native Gβγ partner. Alternatively, an accessory protein (Acc) functioning as a GAP can interact with Gα-GTP to accelerate the deactivation of the signaling pathway **(A)**. Other accessory proteins can bind to the inactive form of Gα-GDP to either facilitate the activity of Gα by increasing the switch from GDP-to-GTP bound Gα subunits (known as GEFs; **B**) or bind one or more Gα_i/o_-GDP subunits to activate other non-canonical signaling pathways (known as GDI; **C**). The last major type of regulation by accessory proteins is a direct interaction with Gβγ in the presence or absence of the associated Gα subunit **(D)**. As an example, an accessory protein (Acc), such as GRK2/3, can bind with Gβγ to distribute the complex to the plasma membrane and phosphorylate an active GPCR to downregulate its activity, or the physical interaction with Gβγ could disrupt the activated Gβγ-dependent signaling by the unbound dimer. It remains unclear whether accessory proteins complexed with Gβγ promote their own unique signal processing. GPCR, G-protein coupled receptor; αβγ, heterotrimeric G-protein α, β, and γ subunits; GDP, guanine dinucleotide phosphate; GTP, guanine trinucleotide phosphate; Acc, accessory protein.

**Figure 2:**
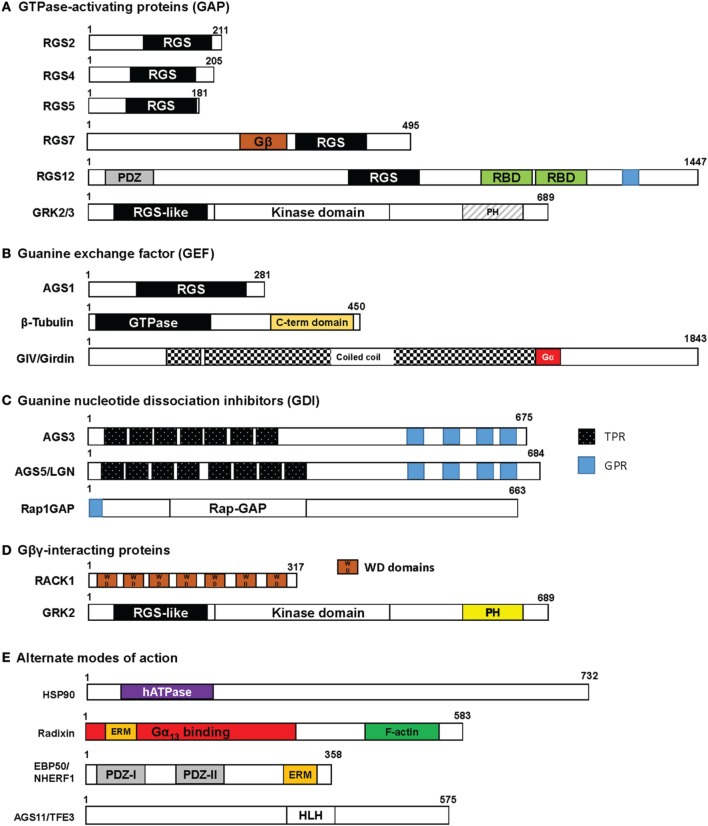
**Protein domain structure in accessory proteins**. Each of the accessory proteins described in this review are drawn with their respective domains using the wild-type protein sizes obtained from a consensus human sequence. The protein structures are categorized by their putative biological roles in regulating G-protein function as shown in Figure 1: **(A)** GAP; **(B)** GEF; **(C)** GDI; and **(D)** Gβγ interaction. G-protein subunits have been determined to interact within the RGS box, Gβ, GPR, PH, or WD domains. In some accessory proteins listed in **(E)** “Alternate mode of action,” the radixin protein has been identified to bind in the N-terminal part of the protein and EBP50 interacts with Gα_q_ in the PDZ domains. The binding sites for HSP90 and AGS11 have yet to be determined. RGS, Regulator of G-protein Signaling box; RBD, Ras binding domain; TPR, tetratricopeptide repeat; GPR, G-protein regulatory motif; WD, WD40 repeat; PDZ, PSD-95/*Drosophila* disk large/ZO-1 domains; PH, pleckstrin homology domain; hATPase, human ATPase; ERM, ezrin-radixin-moesin binding domain; HLH, helix-loop-helix.

In the normal kidney, there are an increasing number of accessory proteins that have been detected using various molecular techniques, but only a few have been described to modulate some aspect of renal tubular function. Figure 1 illustrates the most common modes of action mediated by accessory proteins to control the activation-inactivation cycle of heterotrimeric G-proteins, and are defined in the following categories: (1) GTPase-activating proteins (GAP); (2) guanine exchange factors (GEF); (3) guanine dinucleotide dissociation inhibitors (GDI); and (4) Gβγ-interacting proteins. Figure 2 provides a basic schematic of the protein domain structures for the various accessory proteins.

### GTPase-activating proteins (GAP)

Upon activation of Gα subunits following its binding with GTP, Gα subunits have an intrinsic rate in which GTP can be hydrolyzed back to GDP (Figure 1A). This leads to the re-association with its native Gβγ partners, which inactivates the G-protein signaling output back to control levels (Siderovski and Willard, 2005). Accessory proteins known as GAPs can short circuit the Gα-GTP activation state by accelerating GTP catabolism (Siderovski and Willard, 2005).

#### G-protein coupled receptor kinases (GRK)

This family of seven serine/threonine kinases has been well established to play an integral role in the regulation of GPCR activation (Kamal et al., 2012; Sato et al., 2015). Of the seven GRKs, only five of the non-visual isoforms (GRK2-6) have been identified to some degree in the mouse and human kidneys (Gainetdinov et al., 2000; Felder et al., 2002). Each of the GRK isoforms has a similar protein structure in which the N-terminus contains a RGS homology domain that allows for the binding with Gα_q/11_subunits, with a central core that possesses the kinase activity domain. GRK2/3 is categorized as β-adrenergic kinases, while GRK4-6 can be classified as the GRK4 group (Kamal et al., 2012; Sato et al., 2015). GRK2/3 isoforms possess weak GAP function to desensitize GPCR signaling by selective interaction with Gα_q/11_subunits (Carman et al., 1999b); whereas GRK4 was unable to bind to active Gα_q_ or alter Gα_q_-dependent signaling (Picascia et al., 2004). Another important domain in the C-terminal region of GRK2/3 is the pleckstrin homology (PH) region that can interact directly with Gβγ dimers. This dimeric GRK2/3-Gβγ complex is capable of redistributing to the plasma membrane (Daaka et al., 1997), and spatially orients GRK2/3 for efficient phosphorylation of specific serine and threonine amino acids in the active GPCR. This also enables β-arrestin to be recruited, if needed, to the GPCR and act in concert with GRK2/3 to downregulate the GPCR and terminate G-protein signaling (Kamal et al., 2012).

This type of desensitization phenomenon is observed in the kidney on the dopamine type 1 receptor (D1R) by GRK2 during states of oxidative stress (Banday and Lokhandwala, 2007). Administration of lipoic acid, an antioxidant, could normalize the GRK2 localization and restore the signaling function of the D1R (Banday and Lokhandwala, 2007). Similar findings were confirmed using other methods to control the oxidative stress in the kidneys from hyperglycemic rat models (Trivedi and Lokhandwala, 2005; Marwaha and Lokhandwala, 2006). Other studies have identified GRK2 as a regulator of microtubule assembly (Carman et al., 1999a) and cilia length (Kim et al., 2010), which suggest that GRK may have broader functional implications than the desensitization of GPCR activity.

Two other RGS proteins have shown expression in the kidney, but their role to control renal function under normal conditions needs further investigation:

#### RGS4

RGS4 functions as a GAP to promote deactivation of Gα_q/11_and Gα_i/o_ subunits in a cell-type dependent manner (Bansal et al., 2007). In the mouse kidney, RGS4 was expressed predominantly in the renal vasculature (Siedlecki et al., 2011; Pang et al., 2015).

#### RGS12

RGS12 is the largest RGS family member, which has predominantly been studied for its extra-renal functions in the bone system (Yang et al., 2013; Yuan et al., 2015). Coincidentally, RGS12 was identified in a separate yeast screen used to identify Activator of G-protein Signaling (AGS) proteins, and was also named as AGS6 (Blumer et al., 2005, 2007). Recent immunohistochemical analyses has shown that RGS12/AGS6 is robustly expressed in the endothelial cells of the renal vasculature, and at a lesser intensity throughout the renal tubular epithelia (Lenarczyk et al., 2015). Glomerular staining was absent for RGS12/AGS6. Continued investigation into the role of RGS12 is needed to determine its importance on renal function, particularly with the availability of floxed RGS12 mice (Yang et al., 2013; Yuan et al., 2015).

### Guanine exchange factors (GEF)

Similar to the GEF function exhibited by GPCRs, some intracellular accessory proteins can also bind inactive Gα subunits to modulate the rate in which GDP can be swapped for a molecule of GTP (Siderovski and Willard, 2005). These accessory proteins can stabilize the active Gα-GTP complex to amplify the G-protein signaling output (Figure 1B).

#### Activator of G-protein signaling 1 (AGS1)

AGS1 was the first protein characterized from the AGS family, and its sequence analysis was homologous to dexamethasone-inducible Ras protein, DexRas1 (Cismowski et al., 1999). Studies have determined that AGS1 acts as a GEF by selectively potentiating the binding of GTP to Gα_i_ subunit to promote the activating signaling pathways (Cismowski et al., 1999, 2000, 2001; Tu and Wu, 1999). In the normal mouse and human kidneys, AGS1 mRNA levels were detected at relatively low levels (Kemppainen and Behrend, 1998; Tu and Wu, 1999; Kemppainen et al., 2003), and AGS1 protein was exclusively localized to the proximal tubules in the cortex and outer medulla of the mouse kidneys (Lenarczyk et al., 2015).

#### Tubulin

Tubulin is ubiquitously expressed throughout the kidney, and contributes to a number of biological functions associated with the cytoskeleton, including the maintenance of normal cellular morphology, cell migration, cell division, and intracellular transport mechanisms (Schappi et al., 2014). Polymerization of microtubules consists of a heterodimer of α- and β-tubulin subunits that actively form upon binding to GTP. A number of G-protein α subunits, namely α_s_, α_i_, and α_q_, can directly interact with tubulin to control the dynamic stability of the microtubules. In this scenario, tubulin exhibits a function similar to a GEF, whereby GTP is bound by the β-subunit of tubulin and transferred to Gα subunits to displace for the bound GDP (Schappi et al., 2014). Upon the binding of either Gα_s_ or Gα_i1_ to tubulin, the GTPase activity in tubulin is accelerated leading to microtubule destabilization (Roychowdhury et al., 1999). The intermediary role by which tubulin interacts with G-protein subunits provides some insight as to the mechanism by which GPCR activation transmits information from the external milieu to the cytoskeleton.

### Guanine nucleotide dissociation inhibitors (GDI)

An evolutionarily conserved sequence known as the G-protein regulatory (GPR) motif can bind selectively to inactive Gα_i/o_subunits bound to GDP to exert diverse biological functions within the cell (Siderovski and Willard, 2005) (Figure 1C). Accessory proteins containing one or more of these GPR motifs have been detected in the kidney, and function by maintaining the Gα_i_ subunit in the inactive GDP-bound state to prevent the re-association with its native Gβγ partner. The functional roles of GDI can be quite diverse by controlling mitotic spindle orientation, asymmetric cell division, protein trafficking, autophagic processes, and even regulate ligand-dependent GPCR signaling at the cell membrane (Blumer and Lanier, 2014). All of these functions associated with the GDI have not been identified in the kidney, and the accessory proteins with one or more GPR motifs and expressed in the kidney are listed below:

#### Activator of G-protein signaling 3 (AGS3)

Activator of G-protein Signaling 3 (AGS3) is the third protein identified from yeast screen attempting to identify receptor-independent regulators of G-protein subunit activity (Cismowski et al., 1999, 2001), and is also known as G-protein Signaling Modulator 1 (GPSM1). AGS3/GPSM1 is an evolutionarily conserved protein found in fruit flies to humans, and is a tripartite protein in which there are seven tetratricopeptide repeats at the N-terminus followed by a short linker region connecting four GPR motifs at the C-terminus (Blumer et al., 2002). The expression of AGS3 in the normal rodent kidney is predominantly localized to the collecting ducts in the renal cortex and outer medulla (Nadella et al., 2010; Regner et al., 2011; Kwon et al., 2012; Lenarczyk et al., 2015). Functionally, *Gpsm1*-deficient mice are able to survive post-natal birth without any adverse histological observations in the kidney (Regner et al., 2011; Kwon et al., 2012). *In vitro* studies using proximal tubule and collecting duct epithelial cells demonstrated reduced cell numbers following genetic knockdown of endogenous AGS3 expression, which suggested that AGS3 may play a basal role in controlling renal tubular epithelial cell viability (Nadella et al., 2010; Regner et al., 2011). Further studies are needed to confirm the role of AGS3 in the normal kidney.

#### AGS5

AGS5 is a homolog of AGS3, and is also known by other aliases, including G-protein Signaling Modulator 2 (GPSM2) or more commonly as Leu-Gly-Asn rich protein (LGN). AGS5 has a similar protein structure to AGS3, which includes eight TPR in the N-terminus and four GPR motifs in the C-terminus (Blumer et al., 2002). Unlike AGS3, AGS5 is expressed ubiquitously throughout the mammalian organs, including a high abundance in the kidney (Blumer et al., 2002; Nadella et al., 2010; Regner et al., 2011). AGS5 has been localized to the distal portion of the nephron, specifically the thick ascending limbs of Henle and the collecting ducts (Lenarczyk et al., 2015). Functionally, AGS5/LGN is involved in the establishment of mitotic spindle orientation along the polarity axis of the cell (Blumer et al., 2006). The role of this phenomenon or other functions in the kidney using AGS5/LGN-deficient mice, which are post-natally viable (Konno et al., 2008), remains to be determined.

#### Rap1 GTPase-activating protein (Rap1GAP)

Rap G-proteins are a distinct subfamily of the Ras family of small molecular weight GTPases (Spilker and Kreutz, 2010; Gloerich and Bos, 2011), which can act as a GDI by virtue of the presence of a GPR motif at the N-terminus. A previous study by demonstrated that Rap1GAP could selectively bind to GDP-bound Gα_i_ subunits (Natochin et al., 2001), which was subsequently shown to alter the localization of Rap protein to the cell surface or target for proteosomal degradation (Gloerich and Bos, 2011). Under normal homeostasis, the expression pattern of Rap1GAP in the kidney is minimal (Rubinfeld et al., 1991), and so its biological role remains largely undefined.

### Gβγ-interacting proteins

There are an increasing number of proteins that can interact directly with free Gβγ dimers or complexed as the αβγ heterotrimers to control G-protein signaling (Figure 1D). It is well established that unbound Gβγ can promote G-protein dependent signaling, but the effect of an interaction by accessory proteins with Gβγ on the magnitude, localization and duration of the signaling output needs further investigation.

#### G-protein coupled receptor kinases (GRK)

As discussed earlier, GRK2/3 interacts with the Gβγ subunits with the C-terminal PH domains, which is unique to the GRK2/3 isoforms and not found in the other five GRKs. The newly formed GRK2/3-Gβγ complex will translocate to the plasma membrane for subsequent phosphorylation of the GPCR, which will promote internalization of the GPCR into the cell through an endocytic pathway and trafficked to lysosomes for recycling of the components (Daaka et al., 1997).

#### Receptor for activated protein kinase C 1 (RACK1)

RACK1 was initially cloned from a rat brain cDNA library (Ron et al., 1994), and exhibits diverse cellular functions, including activation by direct binding to the SH2 domain (Chang et al., 1998), and binding to free Gβγ subunits (Chen et al., 2004a,b). This type of protein-protein interaction by RACK1 has been shown to promote translocation of RACK1 to the membrane and partially inhibit Gβ_1_γ_1_-, but not Gα-dependent signaling in cells (Chen et al., 2004a,b). Under normal kidney homeostasis, RACK1 expression was minimal (Padanilam and Hammerman, 1997). However, in isolated pooled pre-glomerular vascular smooth muscle cell lysates, a high abundance of RACK1 protein was detected using Western blot analysis (Cheng et al., 2011). Genetic knockdown of endogenous RACK1 levels in both vascular smooth muscle and glomerular mesangial cells were observed to have a negative impact on cell number due to a decrease in the level of intracellular calcium (Cheng et al., 2011). The physiological effect of RACK1 in the renal tubules and vasculature in the normal kidney *in vivo* remains to be determined.

#### Tubulin

In some instances, tubulin has been shown to bind selectively with Gβγ dimers to promote the assembly of microtubules (Roychowdhury and Rasenick, 1994), but may be isoform-specific (Yan et al., 1996). The site of interaction between Gβγ subunits with tubulin remains to be determined.

### Alternate mechanisms of action by accessory protein-G protein interaction

Upon binding with specific subunits of the heterotrimeric G-protein, the mode of action by which some accessory proteins alter G-protein function have either not been determined, or act in a distinct mechanism outside of the four categories listed earlier. In some cases, accessory proteins may undergo a conformational change to act as a central scaffold to organize signaling protein complexes, or mediate changes in organelle localization within the cell to regulate its function.

#### Regulator of G-protein signaling 2 (RGS2)

Regulator of G-protein Signaling 2 (RGS2) has been shown to control the rate in which water is reabsorbed in mice after dehydration (Zuber et al., 2007), i.e., *Rgs2*^−/−^ mice following dehydration reabsorbed more water during the initial rehydration period compared to the wild-type mice (Zuber et al., 2007). Consistent with its functional role in water reabsorption, RGS2 was localized to nephron segments that were vasopressin V2 receptor-positive and also essential for generating the urine concentrating mechanism, which included the thick ascending limbs of Henle, distal convoluted tubules, and the entire length of the collecting duct (Zuber et al., 2007; Adebiyi, 2014). Glomerular and renal vascular cells were also observed to express RGS2, but minimal expression was detected in the proximal convoluted tubules, or the thin limbs of Henle. Coincidentally, RGS2 expression could be induced following stimulation with arginine vasopressin (AVP) in both isolated thick ascending limbs and cortical collecting ducts (CCD) (Zuber et al., 2007). Moreover, accumulation of cAMP in CCD following AVP stimulation was significantly higher in the absence of RGS2 expression. Although RGS2 was initially identified to selectively bind Gα_q_, the mechanism by which RGS2 regulates cAMP production appeared to be dependent upon direct interaction with either or both Gα_s_ and adenylyl cyclase isoforms (Roy et al., 2003, 2006). Instead of RGS2 functioning as a GAP on Gα_s_ activity, RGS2-Gα_s_ complexes promoted a redistribution of RGS2 to the plasma membrane from the nucleus. This enabled RGS2-Gα_s_ to negatively impact cAMP production by direct interaction with adenylyl cyclases (Roy et al., 2006, 2003). These studies provide some evidence that water reabsorption can be modulated by RGS2 in a mechanism that does not appear to involve the canonical GAP activity on Gα subunits.

#### Transcription factor E3 (TFE3)

Transcription factor TFE3 is a member of the microphthalmia (MiT) family of basic helix-loop-helix-leucine zipper transcription factors (Argani and Ladanyi, 2005), and was recently identified as an AGS protein (AGS11) by virtue of its strong interaction with Gα_16_ and to a lesser extent, Gα_s_ subunits (Sato et al., 2011). A previous study demonstrated that renal tubular phosphate reabsorption may be regulated by TFE3 (Miyamoto and Itho, 2001). In mice fed a low phosphate diet, NPT2 expression was elevated. Truncated forms of TFE3 induced NPT2 expression by directly binding to phosphate response elements (PRE) using nuclear extracts isolated from mouse proximal tubular cells. Further studies are needed to confirm whether the mode of action by TFE3/AGS11 is through the altered nuclear translocation of G-protein subunits in the kidney, but there is a previous study using cardiomyocytes that an AGS11-Gα_16_ complex preferred nuclear localization and was an essential step in the enhancement of cardiomyocyte transcriptional activation (Sato et al., 2011).

#### Radixin

Radixin is a member of the ezrin-radixin-moesin (ERM) family of actin-binding proteins, which can function as a scaffold protein between filamentous actin (F-actin) and membrane associated proteins to regulate signal transduction pathways for fluid and electrolyte balance and cytoskeletal remodeling. Radixin localizes to the renal corpuscles and the brush border of the proximal tubules (Ingraffea et al., 2002). Raxidin interacts selectively with the active form of Gα_13_-GTP complexes, but not Gα_12_, and promotes radixin to undergo a conformational change that unmasks a high affinity binding site for F-actin at the C-terminus (Vaiskunaite et al., 2000). Functionally, the specific role for radixin-Gα_13_ has not been thoroughly investigated in the kidney, and genetic removal of radixin in mice produces viable offspring that exhibit relatively normal renal phenotype (Morales et al., 2004). Considering that other members of the ERM family, particularly ezrin, have high homology (~75%) to the human form of radixin, additional genetic knockouts of the ERM family may be warranted to fully expose the biological role of radixin in the kidney.

#### EBP50 (ezrin-radixin-moesin-binding phosphoprotein 50)

Another scaffold protein associated with the ERM family is EBP50, which was initially identified as a protein that can bind to the N-terminal domain of ezrin or moesin (Reczek et al., 1997). EBP50 is the human homolog of the rabbit Na^+^–H^+^ exchanger regulatory factor 1 (NHERF1). In the kidney, the distribution of mRNA expression for EBP50/NHERF1 was localized to the glomerulus, proximal tubules, thick ascending limbs of Henle and CCD by qualitative RT-PCR, but not the thin descending limbs of Henle or the medullary collecting ducts (Weinman et al., 1995). Immunohistochemistry using a specific antibody to NHERF1 demonstrated exclusive localization to the apical membrane in the proximal tubules (Miyazaki et al., 2005). EBP50/NHERF1 can bind to a multitude of proteins due to the presence of two interspaced PDZ domains in the central compartment of the protein. The GTP-bound form of Gα_q_ was demonstrated to interact with both PDZ domains in EBP50/NHERF1 (Rochdi et al., 2002), and interferes with downstream activation of phospholipase C-β1 (PLC-β1). Studies in EBP50 knockout mice demonstrated that the absence of EBP50 resulted in a marked reduction in the localization of ERM proteins to their typical site at the brush border membrane in proximal tubules (Morales et al., 2004), but whether abnormalities in G-protein signaling is associated with this defect in compartmentalization is not well established.

#### Heat shock protein 90 (HSP90)

Heat shock protein 90 (HSP90) is a molecular chaperone protein that can interact with a large host of substrate proteins known as “clients.” Of the various heterotrimeric G-protein subunits, Gα_12_, but not Gα_13_ subunits (Vaiskunaite et al., 2001), as well as Gβγ (Inanobe et al., 1994) have been shown to selectively bind with HSP90. To date, the precise site of interaction with the G-protein subunits with HSP90 remains to be determined. In the normal kidney, HSP90 is expressed at a low level (Barrera-Chimal et al., 2014; Smithline et al., 2014), and can be detected in the distal tubule and collecting duct epithelial cells (Satoh et al., 1994), which are common sites of Gα_12_ subunit localization (Zheng et al., 2003; Boucher et al., 2012). Under normal conditions, HSP90 acts as a surveillance system to ensure proper conformational protein production and function, and its expression is induced during cellular stress (Burrows et al., 2004; Whitesell and Lin, 2012). The role of HSP90 in the kidney under basal conditions is not well defined, but there is evidence that blockade of HSP90 function could reduce glomerular filtration rate (GFR) through a nitric oxide-dependent mechanism (Ramirez et al., 2008).

## Accessory proteins in kidney injury and disease

### Cystic kidney disease

Cystic kidney disease is believed to be associated with a wide range of genetic and environmental factors (Bisceglia et al., 2006), and the most common form in humans is known as autosomal dominant polycystic kidney disease (ADPKD). In ADPKD, a mutant form of polycystin-1 (PC-1) protein is produced leading to aberrant signaling pathways and phenotypic changes within the renal tubular epithelial cell. These changes lead to the transformation of the normal tubular epithelial cell to exhibit a cystic phenotype resulting in the formation of numerous fluid-filled sacs within the kidney.

PC-1 is an atypical G-protein coupled receptor (GPCR) due to its 11 transmembrane-spanning regions. The C-terminal tail of PC-1 is highly conserved across species, and contains a 20 amino acid G-protein activation domain that interacts and increases the GTPase activity of various isotypes of G-protein α subunits, including Gα_12/13_(Parnell et al., 1998, 2002; Yu et al., 2011), Gαi_1−3_(Parnell et al., 1998, 2002) and Gα_q_ (Parnell et al., 1998). The effector systems activated by the Gα-PC-1 complex include c-Jun N-terminal kinase (JNK) and the AP-1 transcription factor (Parnell et al., 2002) through Gα_12_ (Yu et al., 2010, 2011), and Gα_*i*_ interaction promotes increased pool of Gβγ dimers leading to heteromeric polycystin-1/polycystin-2 channel and G-protein inward rectifying K^+^ (GIRK) activity (Delmas et al., 2002). Binding of G-protein subunits can be antagonized by polycystin-2 (PC-2), which is known to interact with the C-terminal end of PC-1, and control G-protein signaling output (Delmas et al., 2002). This provides the opportunity for other proteins in close proximity to the C-terminal tail of PC-1 to control G-protein subunit binding and modulate G-protein dependent signal transduction activity.

#### Regulator of G-protein signaling 7 (RGS7)

RGS7, a rapidly degraded protein, was found to interact with the C-terminal tail of PC-1 to redistribute to the plasma membrane and prolong its stability (Kim et al., 1999). RGS7 belongs to a subfamily of RGS proteins, which can selectively bind to Gα_o_ and Gα_q_ subunits to accelerate the intrinsic GTPase-activating function to catalyze the degradation of GTP to GDP (Hollinger and Hepler, 2002). Subsequently, it was determined that RGS7 contains a G-protein gamma-like (GGL) domain that can selectively interact with the β5-subunit (Snow et al., 1998). Unlike the G-protein α subunits, RGS7 was believed to interact at the coiled coil domain (L4215-R4249) in PC-1 similar to the site that binds to PC-2. It is not known whether RGS7 or other accessory proteins would compete with PC-2 to control the activity of the PC-2 ion channel. QTL analysis in the BPK mouse model of ARPKD suggested that the region in *Chr 1* containing the RGS7 locus was considered to be a genetic modifier of cystic disease (Guay-Woodford et al., 2000). However, there have not been subsequent genetic studies to confirm their functional relevance to regulate cystic disease progression. Moreover, the sites of RGS7 localization in the kidney remains to be fully determined, particularly its presence in cystic epithelial cells.

#### Activators of G-protein signaling (AGS)

Subsequently, accessory proteins in the Activators of G-protein Signaling (AGS) family have been identified to control the transformation of renal tubular epithelial cells toward a cystic phenotype in animal models. AGS proteins are another large family of receptor-independent G-protein subunit regulators and exhibit distinct mechanisms of action by binding directly to Gα or Gβγ subunits (Blumer et al., 2005, 2007). There is histological evidence that several of the AGS proteins, including AGS3, AGS5, and AGS11, express in renal cystic tubular epithelial cells (Chen et al., 2008; Nadella et al., 2010; Kwon et al., 2012; Lenarczyk et al., 2015). No expression of AGS1 and AGS6 was detected in renal cystic epithelial cells (Lenarczyk et al., 2015).

#### Activator of G-protein signaling 3 (AGS3)

Activator of G-protein Signaling 3 (AGS3) [or G-protein Signaling Modulator 1 (GPSM1)] mRNA and protein expression was aberrantly high in cystic collecting duct epithelial cells from multiple orthologous and non-orthologous rodent models of autosomal recessive (ARPKD) and ADPKD (Nadella et al., 2010; Kwon et al., 2012; Lenarczyk et al., 2015). Functional loss of full-length AGS3/GPSM1 protein expression in the *Pkd1*^*V*/*V*^ mouse, a model of ADPKD, demonstrated an acceleration of cystic disease progression in the kidneys (Kwon et al., 2012). The signaling pathways affected by alterations in AGS3/GPSM1 protein expression have not been fully described in the context of cystic epithelial cells, but there is compelling evidence in other cell lines that AGS3/GPSM1 functions as a guanine dinucleotide dissociation inhibitor (GDI) due to the presence of four GPR motifs (Cismowski et al., 1999; Takesono et al., 1999). The GPR motifs interact directly with one or more Gα_i/o_ subunits bound to GDP (12–14), which prevents the re-association with Gβγ subunits and prolong Gβγ-dependent signaling as one mode of action (Nadella et al., 2010; Regner et al., 2011; Kwon et al., 2012). Consistent with this mechanism of action, over-expression of AGS3/GPSM1 in the absence of a Gβγ scavenger was observed to facilitate an increase of heteromeric PC-1/PC-2 ion channel activity (Kwon et al., 2012). Alternatively, there is recent evidence that AGS3/GPSM1 interfaces with activated insulin growth factor 1 receptor (IGF-1R) at the cell membrane to control Gβγ-dependent resorption of the cilia (Yeh et al., 2013). Further studies are needed to fully understand the implications of the AGS3-Gβγ signaling in the control of PC-1/PC-2 ion channel function and cilia dynamics in renal tubular epithelial cells.

#### Activator of G-protein signaling 5 (AGS5)

Similar to AGS3/GPSM1, AGS5 also known more commonly as LGN, was detected in the distal tubular epithelial cells in ADPKD kidneys (Lenarczyk et al., 2015). Unlike AGS3/GPSM1, however, the protein levels of AGS5/LGN were not markedly changed during the progression of cystic disease (Nadella et al., 2010). An *in vitro* study using MDCK epithelial cells demonstrated abnormal formation of cysts (Zheng et al., 2010; Xiao et al., 2012), but the role for AGS5/LGN in cystic kidney disease has yet to be studied using animal models.

#### Activator of G-protein signaling 11 (AGS11)

In Birt-Hogg-Dubé (BHD) syndrome, which is a rare genetic disease caused by mutations in the folliculin (*FLCN*) gene, renal cystic epithelial cells exhibit increased nuclear localization of AGS11/TFE3 (Chen et al., 2008). Further studies are needed to confirm whether Gα_16_ plays a role, if any, in the nuclear localization through a dimeric interaction with AGS11/TFE3 in this genetic disease.

#### Heat shock protein 90 (HSP90)

In mouse *Pkd1*^−/−^ and human ADPKD kidneys, the expression of HSP90 was markedly elevated in the cystic tubular epithelial cells compared to wild-type control kidneys (Seeger-Nukpezah et al., 2013). Blockade of HSP90 using a pharmacological inhibitor reduced cystic disease progression in the kidney (Seeger-Nukpezah et al., 2013) and liver (Smithline et al., 2014). At present, however, the role of the HSP90-Gα_12_ in animal models of ADPKD remains to be determined, but there is evidence that Gα_12_ can directly interact with PC-1 (Yu et al., 2011) and HSP90 can be associated with the cilia (Prodromou et al., 2012). Activity of Gα_12_-dependent apoptosis was associated with the level of PC-1 expression i.e., over-expression of PC-1 promotes Gα_12_ activity whereas knockdown of PC-1 leads to reduced Gα_12_ activity (Vaiskunaite et al., 2001). The regulation of Gα_12_ activation by PC-1 may in part include HSP90 at the level of the cilia, so further studies would be necessary to confirm the relationship between PC-1/ Gα_12_/HSP90 in the context of cystic disease progression.

#### Tubulin

Microtubules are considered to be a major constituent of the cell, particularly in the cilia and centrosomes, and these cellular features that have been implicated in the pathogenesis of cystic disease (Woo et al., 1997). In animal models of polycystic kidney disease, drugs that promoted microtubule assembly were capable of reducing the cystic disease progression and significantly extended the survivability of the cystic mice (Woo et al., 1997). The mechanism by which G-protein subunits interact with tubulin to control the dynamics of microtubule assembly during cystic disease remains to be fully characterized. Loss of cilia increases the acetylation of α-tubulin, but the total pool of β-tubulin remains unchanged (Berbari et al., 2013). Alternatively, abnormalities in the mitotic spindle orientation may be involved in the pathogenesis of cystic kidney, but not in all genetic forms of PKD (Fischer et al., 2006; Saburi et al., 2008; Luyten et al., 2010; Nishio et al., 2010). As a potential mechanism of action, accessory proteins acting as GDI, such as AGS3 or AGS5/LGN, which are known to control mitotic spindle orientation may be perturbed in cystic epithelial cells leading to their misalignment from the normal axis. Further studies focusing on the importance of tubulin interaction with G-protein subunits are crucial in elucidating the etiology of cystogenesis using orthologous models of PKD in the absence or over-expression of specific accessory proteins.

### Ischemia-reperfusion injury

Acute kidney injury (AKI) is a major clinical problem observed in hospitalized children and adults, and is commonly associated with hypoperfusion of the kidney and sepsis (Basile et al., 2012; Ferenbach and Bonventre, 2015). Imbalances in the regulation of G-protein signaling is known to partially impact the full recovery of the damaged tubular epithelia, and this incomplete repair of the kidney can lead to chronic kidney abnormalities and loss of renal function. The following accessory proteins have been described to interact with G-protein subunits and regulate renal function:

#### Regulator of G-protein signaling 4 (RGS4)

In RGS4-deficient mice, renal tubular epithelial cells were highly susceptible to ischemia-reperfusion injury (IRI). Smooth muscle cell (SMMC)-specific knockout of RGS4 in mice exhibited increased mortality following bilateral IRI compared to the control SMMC-Cre mice. Conversely, transgenic mice over-expressing RGS4 were protected from IRI (Pang et al., 2015). The loss of RGS4 following ischemia was associated with a marked reduction in total renal blood flow (Siedlecki et al., 2011), and reduced inflammatory cytokine production from macrophages (Pang et al., 2015). Early after reperfusion following ischemia, RGS4 competed with downstream effectors for Gα_*q*_ subunits activated by ANGII to prevent the normal induction of Regulated on Activation, Normal T cell Expressed and Secreted (RANTES) following angiotensin II-stimulated in isolated vascular smooth muscle cells (Pang et al., 2015).

#### Activator of G-protein signaling 1 (AGS1)

Following bilateral renal IRI, AGS1 mRNA levels were markedly induced by five-fold after 24 h compared to time-control sham rats (Lenarczyk et al., 2015). There is recent studies demonstrating that dexamethasone administration has a protective effect on the kidney following renal IRI in part by inducing the expression of anti-apoptotic protein, serum and glucocorticoid regulated kinase-1 (SGK-1) (Rusai et al., 2013), or preventing the activation of PI3K/Akt signaling on the inflammatory response system (Zhang et al., 2013). It is intriguing to consider that dexamethasone may also promote AGS1 induction as an alternate mode of action to provide protection to the kidney following renal IRI. Further studies in genetically modified mice will be necessary to confirm the role of AGS1 in the kidney.

#### Activator of G-protein signaling 3 (AGS3)

Following ischemia-reperfusion injury, AGS3/GPSM1 protein expression was selectively induced in the recovering outer medullary proximal tubular epithelial cells (Regner et al., 2011; Lenarczyk et al., 2015). A genetic deletion in exon 2 of the *Gpsm1* gene resulted in an absence of full-length AGS3/GPSM1 protein expression, which caused tubular epithelial cell damage to persist following IRI compared to the wild-type mice (Regner et al., 2011). The inability of the renal proximal tubular epithelial cells to recover in the *Gpsm1*^−/−^ mice following IRI was partially associated with a decreased number of Ki67-positive cells. Endogenous knockdown of AGS3/GPSM1 in normal rat kidney (NRK-52E) epithelial cells reduced cell number, but the signaling mechanisms regulated by AGS3/GPSM1 during conditions that simulate acute renal cell injury is largely undescribed. As described earlier, AGS3/GPSM1 is capable of sequestering Gα_i_ subunits to prevent the inactivation of Gβγ signaling (Nadella et al., 2010; Regner et al., 2011; Kwon et al., 2012). However, there is evidence to suggest that AGS3/GPSM1 regulates macroautophagy, an emerging mode of action that promotes tubular epithelial cell recovery following AKI, by direct interaction with Gα_i3_ subunits (Pattingre et al., 2003, 2004; Ghosh et al., 2010; Groves et al., 2010). Alternatively, we could speculate that AGS3/GPSM1 influences signal transduction pathways through other protein-protein interactions, such as liver kinase B1 (LKB1) (Blumer et al., 2003), which is a serine/threonine kinase known to regulate AMPK signaling during states of energetic stress (Alexander and Walker, 2011). Further studies are needed to define the signaling pathways modulated by AGS3/GPSM1 to control cell survival during states of energy depletion and recovery in renal tubular epithelial cells.

#### Receptor for activated C kinase (RACK1)

A marked temporal induction of RACK1 exclusively in the damaged and recovering proximal tubular epithelial cells was observed during the initial 7 day period after renal ischemia-reperfusion injury (Padanilam and Hammerman, 1997). The functional consequences for RACK1 during tubular epithelial cell recovery remain unknown, since mice with a global deficiency in RACK1 expression exhibit premature lethality (Volta et al., 2013). Selective deletion of RACK1 in the kidneys, and possibly to the renal tubular system, will be necessary to eventually define the importance of RACK1 in the kidney during acute kidney injury.

#### Heat shock protein 90 (HSP90)

HSP90 expression was induced in the S3 segment of the proximal tubules following IRI (Morita et al., 1995). Pharmacological inhibition of HSP90 reduced the tubular epithelial cell damage following ischemia-reperfusion injury, and was associated with an induction of other heat shock protein 70 and 27, which can activate cytoprotective pathways (Harrison et al., 2008). Conversely, over-expression of HSP90α/β in the kidney was partially effective in reducing tubular epithelial cell injury following ischemia-reperfusion injury, which was associated with the re-establishment in the coupling of endothelial nitric oxide synthase (eNOS) (Barrera-Chimal et al., 2014). To date, the role by which G-protein signaling through the dimeric interaction between HSP90 and Gα_12_ or Gβγ subunits to control epithelial cell damage and recovery following AKI needs further investigation.

### Renal vascular effects and blood pressure regulation

The kidney is considered as a major controller of long-term blood pressure regulation due to its infinite ability to excrete fluid and electrolytes (Cowley, 1992). Changes in either tubular or blood flow distribution are involved in the ability of the kidneys to control blood pressure in mammals. To date, there is a paucity of data regarding the role of accessory proteins regulating renal function to control long-term blood pressure.

#### Regulator of G-protein signaling 2 (RGS2)

In *Rgs2*-deficient mice (Hercule et al., 2007; Gurley et al., 2010), elevated mean arterial blood pressures have been measured compared to wild-type RGS2 littermates, and that the differences in the magnitude in blood pressures were further exaggerated during the night (Hercule et al., 2007). To assess the importance of the kidney to mediate the increased blood pressure, an elegant cross-transplantation study was performed in which *Rgs2*^−/−^ kidneys were transplanted into normal C57Bl/6 mice (Gurley et al., 2010). An increase in mean arterial pressure of 7–10 mmHg was measured in the renal *Rgs2*^−/−^ mice compared to the wild-type C57Bl/6 mice or the systemic *Rgs2*^−/−^ mice transplanted with normal *Rgs2*^+/+^ kidneys. Following stimulation with various vasoconstrictors, including angiotensin II (ANGII), phenylephrine, and endothelin-1, the sensitivity of renal interlobar arteries from *Rgs2*^−/−^ mouse kidneys were significantly higher compared to the same vessels from wild-type mouse kidneys (Hercule et al., 2007). These data would suggest that RGS2 could control blood pressure levels through a renal-dependent mechanism that was associated with either changes in vascular tone and/or tubular reabsorption of fluid and electrolytes.

#### RACK1

Expression and localization of RACK1 was differentially regulated in spontaneously hypertensive rat (SHR) pre-glomerular vascular smooth muscle cells compared to their wild-type Wistar Kyoto (WKY) rat counterparts (Cheng et al., 2013). RACK1 interaction with Gβγ was proposed to redistribute the complex to the membrane and function as a scaffold to bridge other signaling molecules (i.e., protein kinase C and phospholipase C) for downstream activation of diacylglycerol and calcium (Cheng et al., 2013). This effect was dependent upon activation of neuropeptide receptors to liberate Gβγ from its Gα_i_ subunit, and was substantially more active in the SHR vs. WKY vascular cells to promote proliferation (Cheng et al., 2013). The physiological impact on renal vascular resistance and overall blood pressure regulation remains to be determined.

### Renal fibrosis

Renal fibrosis is a classic phenotype in the progression of many types of renal disease, and occurs when the deposition of extracellular matrix components begin to replace the normal tubular epithelial cells (Chevalier et al., 2009; Meng et al., 2014).

#### RGS2

Using a rodent model of unilateral ureteral obstruction (UUO), which can simulate progressive renal fibrosis, RGS2 was shown to beneficially slow the progression of kidney fibrosis (Jang et al., 2014). In this study, the authors demonstrated that RGS2 protein levels were markedly increased after 5 days following the UUO procedure with no difference in Gα_q_ levels. Moreover, there was increased production of both fibrotic markers, α-smooth muscle action and collagen, and inflammatory markers, F4/80, Ly6G, myeloperoxidase, and CXCR4, in the UUO-treated kidneys from the *Rgs2*-deficient mice. Further *in vitro* studies showed that over-expression of RGS2 could block the ANGII-mediated activation of ERK1/2 signaling and prevent the increase in CXCR4 expression. This study provides evidence that RGS2 attenuated the onset of renal fibrosis in part by accelerating the deactivation of the angiotensin type 1 receptor (AT1R)-mediated signaling of the pro-fibrogenic and inflammatory systems (Jang et al., 2014).

### Glomerular injury

The glomerulus is an important anatomic structure in the kidney that consists of a tuft of capillaries that is encapsulated by Bowman's capsule, and other important distinct cell types, including the podocytes, mesangial cells, and basement membrane, that are involved in controlling the permeability and selectivity parameters for filtration of the blood into the tubular lumen (Alpern et al., 2013). Injury to the glomerulus by genetic or environmental factors can lead to pathologies that promote abnormal protein excretion and possibly, chronic renal failure. Emerging new studies have been described regarding the potential role of accessory proteins following injury to the glomerulus.

#### Gα interacting, vesicle-associated protein (GIV)/Girdin

GIV/Girdin was initially identified to interact with G-protein α_i3_/s subunit, and subsequently characterized as a GEF (Garcia-Marcos et al., 2015) exhibiting diverse biological effects, including cell migration, autophagy, survival and intracellular protein trafficking (Garcia-Marcos et al., 2015). In the kidney, however, the gene expression of GIV/Girdin under normal homeostasis is relatively sparse. During glomerular damage, the production of a copious amount of GIV/Girdin can be induced selectively within the podocytes (Wang et al., 2015), and this regulates the activation of the PI3K/Akt survival pathway. Ultimately, this provides enhanced protection from the injury stimuli mediated by the chemical administration of puromycin aminonucleoside (PAN) (Wang et al., 2015).

#### Rap1 GTPase-activating protein (Rap1GAP)

In transgenic mice expressing human immunodeficiency virus type 1 (HIV-1), the expression of Rap1GAP was induced selectively in renal glomerular podocytes (Potla et al., 2014). Increased expression of Rap1GAP was also detected in kidney samples obtained from patients with focal and segmental glomerulosclerosis (FSGS) (Potla et al., 2014). The induction of Rap1GAP was associated with the podocyte dysfunction after injury by impairing the mechanisms involved in the activation of β1-integrin adhesive function. This ultimately promoted podocyte detachment and exacerbated cellular death.

#### Regulator of G-protein signaling 2 (RGS2)

In children with glomerulonephritis, urotensin II (UII) was detected in distinct compartments of the glomerulus (Balat et al., 2007), but its role is not well defined. Recent evidence in glomerular mesangial cells suggests that RGS2 can reduce the ability of urotensin II (UII) to increase intracellular calcium and contraction (Adebiyi, 2014), and demonstrates RGS2 as a negative regulator of G-protein activity following activation of the UII/UII receptor axis in the glomerulus.

### Renal cancer

Of the known forms of kidney cancer, the most prevalent is renal cell carcinoma (RCC), which accounts for more than 80% of the diagnosed cases and is recognized as the ninth most common form of cancer in the world (Walker, 1998; Koul et al., 2011; Jonasch et al., 2014). RCC is generally refractory to conventional therapies involving drugs and radiation, and so tumor management is largely treated by partial nephrectomy of the affected area. G-protein dependent signaling has been implicated as a contributing factor in the pathogenesis of renal cancer (Knieke et al., 2009; Jonasch et al., 2014; Ma et al., 2015). Recent studies have shown that accessory proteins, TFE3/AGS11, Rap1GAP, and RGS5, were associated with differential expression patterns and localization within the oncogenic cells compared to the normal renal parenchyma.

#### AGS11/TFE3

Xp11 translocation RCC is a group of neoplasms characterized by translocations involving a genomic breakpoint at Xp11.2. In these subtypes of renal cancer, genomic DNA containing either AGS11/TFE3 (Meloni et al., 1993; Weterman et al., 1996a,b) or AGS12/TFEB (Kuiper et al., 2003) are abnormally translocated or inverted into other genes (Weterman et al., 2000, 2001; Mathur and Samuels, 2007), and this is associated with nuclear localization of AGS11/TFE3 and to a lesser extent, AGS12/TFEB. Under normal conditions, the protein localization of AGS11/TFE3 is either observed in predominantly in the cytoplasm or weakly in the nucleus (Hong et al., 2010). Because of this shift in subcellular location to the nucleus, AGS11/TFE3 is considered to be one of the diagnostic biomarkers for specific subtypes of renal cell carcinoma. The mechanism by which G-protein signaling and nuclear translocation is regulated during translocation RCC remains to be determined, but there was some evidence that AGS11-Gα_16_ accumulation in the nucleus promoted robust cardiomyocyte expression of claudin 14, a cell junction protein (Sato et al., 2011). There are an increasing number of studies investigating the expression of specific claudin isoforms as pathogenic biomarkers of various renal cancers (Virman et al., 2014; Men et al., 2015). The role of AGS11/TFE3 in this transcriptional activation process may warrant further evaluation in this specific type of renal cancer.

### Rap1GAP

In renal carcinoma, the Rap1GAP protein levels were reduced leading to increased cellular invasion (Kim et al., 2012). This is consistent with findings in human thyroid tumors where downregulation of Rap1GAP expression promoted aberrant cell migration and invasion, which could be restored back to normal upon re-expression of RAP1GAP (Tsygankova et al., 2007). Further studies are needed to confirm the signaling pathways perturbed by the changes in Rap1GAP function in the renal oncogenic cells to fully appreciate the impact of these observational findings.

### RGS5

In human RCC, RGS5 was detected specifically in the tumor vessels, but not in the tumor cells or in the normal capillaries within the renal parenchyma (Furuya et al., 2004). These observational studies provide evidence that selective RGS5 signal modulation in tumor vasculature may play a role in exacerbating not only human RCC progression, but other types of cancers (Silini et al., 2012).

## Conclusions and future perspectives

Although the kidney has a well-documented history of canonical GPCR-dependent signaling in the renal vascular, glomerular and tubular system, the role of accessory proteins on G-protein function has only begun to emerge within the last few years. As described in this review, the mechanisms associated with the regulation of heterotrimeric G-protein signaling have continued to expand far beyond the control by cell surface receptors activation by their ligands. G-protein signaling through the actions of accessory proteins can modulate canonical GPCR signaling, but also enable additional levels of regulation in which the G-protein signaling can be controlled for its magnitude, duration, and possibly the site of action. Because of the unique cellular composition within not only each region of the kidney (i.e., cortex, outer medulla, and inner medulla), but also within each segment of the nephron, the expression and localization patterns of GPCRs, G-protein subunits, and other accessory proteins demonstrate the enormous task that remains to completely elucidate the mechanisms involved in the control of G-protein signaling within each distinct cell type and segment in the kidney. Further description of these accessory proteins will enable us to fully appreciate the biological significance of heterotrimeric G-protein regulation during normal and diseased states of the kidney.

### Conflict of interest statement

The author declares that the research was conducted in the absence of any commercial or financial relationships that could be construed as a potential conflict of interest.

## References

[B1] Adebiyi A. (2014). RGS2 regulates urotensin II-induced intracellular Ca2+ elevation and contraction in glomerular mesangial cells. J. Cell. Physiol. 229, 502–511. 10.1002/jcp.24470 24105430 PMC11250777

[B2] Alexander A. Walker C. L. (2011). The role of LKB1 and AMPK in cellular responses to stress and damage. FEBS Lett. 585, 952–957. 10.1016/j.febslet.2011.03.010 21396365

[B3] Alpern R. J. Caplan M. J. Moe O. W. (2013). Seldin and Giebisch's The Kidney: Physiology & Pathophysiology, 5th Edn. Waltham, MA: Academic Press.

[B4] Argani P. Ladanyi M. (2005). Translocation carcinomas of the kidney. Clin. Lab. Med. 25, 363–378. 10.1016/j.cll.2005.01.008 15848741

[B5] Balat A. Karakök M. Yilmaz K. Kibar Y. (2007). Urotensin-II immunoreactivity in children with chronic glomerulonephritis. Ren. Fail. 29, 573–578. 10.1080/08860220701392108 17654320

[B6] Banday A. A. Lokhandwala M. F. (2007). Oxidative stress reduces renal dopamine D1 receptor-Gq/11alpha G protein-phospholipase C signaling involving G protein-coupled receptor kinase 2. Am. J. Physiol. Renal Physiol. 293, F306–F315. 10.1152/ajprenal.00108.2007 17459951

[B7] Bansal G. Druey K. M. Xie Z. (2007). R4 RGS proteins: regulation of G-protein signaling and beyond. Pharmacol. Ther. 116, 473–495. 10.1016/j.pharmthera.2007.09.005 18006065 PMC2156173

[B8] Barrera-Chimal J. Pérez-Villalva R. Ortega J. A. Uribe N. Gamba G. Cortés-González C. (2014). Intra-renal transfection of heat shock protein 90 alpha or beta (Hsp90alpha or Hsp90beta) protects against ischemia/reperfusion injury. Nephrol. Dial. Transplant. 29, 301–312. 10.1093/ndt/gft415 24166465

[B9] Basile D. P. Anderson M. D. Sutton T. A. (2012). Pathophysiology of acute kidney injury. Compr. Physiol. 2, 1303–1353. 10.1002/cphy.c110041 23798302 PMC3919808

[B10] Berbari N. F. Sharma N. Malarkey E. B. Pieczynski J. N. Boddu R. Gaertig J.. (2013). Microtubule modifications and stability are altered by cilia perturbation and in cystic kidney disease. Cytoskeleton 70, 24–31. 10.1002/cm.21088 23124988 PMC3552319

[B11] Bisceglia M. Galliani C. A. Senger C. Stallone C. Sessa A. (2006). Renal cystic diseases: a review. Adv. Anat. Pathol. 13, 26–56. 10.1097/01.pap.0000201831.77472.d3 16462154

[B12] Blumer J. B. Lanier S. M. (2014). Activators of G protein signaling exhibit broad functionality and define a distinct core signaling triad. Mol. Pharmacol. 85, 388–396. 10.1124/mol.113.090068 24302560 PMC3935153

[B13] Blumer J. B. Bernard M. L. Peterson Y. K. Nezu J. Chung P. Dunican D. J.. (2003). Interaction of activator of G-protein signaling 3 (AGS3) with LKB1, a serine/threonine kinase involved in cell polarity and cell cycle progression: phosphorylation of the G-protein regulatory (GPR) motif as a regulatory mechanism for the interaction of GPR motifs with Gi alpha. J. Biol. Chem. 278, 23217–23220. 10.1074/jbc.C200686200 12719437

[B14] Blumer J. B. Chandler L. J. Lanier S. M. (2002). Expression analysis and subcellular distribution of the two G-protein regulators AGS3 and LGN indicate distinct functionality. Localization of LGN to the midbody during cytokinesis. J. Biol. Chem. 277, 15897–15903. 10.1074/jbc.M112185200 11832491

[B15] Blumer J. B. Cismowski M. J. Sato M. Lanier S. M. (2005). AGS proteins: receptor-independent activators of G-protein signaling. Trends Pharmacol. Sci. 26, 470–476. 10.1016/j.tips.2005.07.003 16084602

[B16] Blumer J. B. Kuriyama R. Gettys T. W. Lanier S. M. (2006). The G-protein regulatory (GPR) motif-containing Leu-Gly-Asn-enriched protein (LGN) and Gialpha3 influence cortical positioning of the mitotic spindle poles at metaphase in symmetrically dividing mammalian cells. Eur. J. Cell Biol. 85, 1233–1240. 10.1016/j.ejcb.2006.08.002 17000024

[B17] Blumer J. B. Smrcka A. V. Lanier S. M. (2007). Mechanistic pathways and biological roles for receptor-independent activators of G-protein signaling. Pharmacol. Ther. 113, 488–506. 10.1016/j.pharmthera.2006.11.001 17240454 PMC1978177

[B18] Boucher I. Yu W. Beaudry S. Negoro H. Tran M. Pollak M. R.. (2012). Galpha12 activation in podocytes leads to cumulative changes in glomerular collagen expression, proteinuria and glomerulosclerosis. Lab. Invest. 92, 662–675. 10.1038/labinvest.2011.198 22249312 PMC3338890

[B19] Burrows F. Zhang H. Kamal A. (2004). Hsp90 activation and cell cycle regulation. Cell Cycle 3, 1530–1536. 10.4161/cc.3.12.1277 15539946

[B20] Carman C. V. Lisanti M. P. Benovic J. L. (1999a). Regulation of G protein-coupled receptor kinases by caveolin. J. Biol. Chem. 274, 8858–8864. 10.1074/jbc.274.13.8858 10085129

[B21] Carman C. V. Parent J. L. Day P. W. Pronin A. N. Sternweis P. M. Wedegaertner P. B.. (1999b). Selective regulation of Galpha(q/11) by an RGS domain in the G protein-coupled receptor kinase, GRK2. J. Biol. Chem. 274, 34483–34492. 10.1074/jbc.274.48.34483 10567430

[B22] Chang B. Y. Conroy K. B. Machleder E. M. Cartwright C. A. (1998). RACK1, a receptor for activated C kinase and a homolog of the beta subunit of G proteins, inhibits activity of src tyrosine kinases and growth of NIH 3T3 cells. Mol. Cell. Biol. 18, 3245–3256. 9584165 10.1128/mcb.18.6.3245 PMC108906

[B23] Chen J. Futami K. Petillo D. Peng J. Wang P. Knol J.. (2008). Deficiency of FLCN in mouse kidney led to development of polycystic kidneys and renal neoplasia. PLoS ONE 3:e3581. 10.1371/annotation/136385d5-b241-4ecc-b31a-6dea3ebf3bc4 18974783 PMC2570491

[B24] Chen S. Dell E. J. Lin F. Sai J. Hamm H. E. (2004a). RACK1 regulates specific functions of Gbetagamma. J. Biol. Chem. 279, 17861–17868. 10.1074/jbc.M313727200 14963031

[B25] Chen S. Spiegelberg B. D. Lin F. Dell E. J. Hamm H. E. (2004b). Interaction of Gbetagamma with RACK1 and other WD40 repeat proteins. J. Mol. Cell. Cardiol. 37, 399–406. 10.1016/j.yjmcc.2004.04.019 15276010

[B26] Cheng D. Zhu X. Barchiesi F. Gillespie D. G. Dubey R. K. Jackson E. K. (2011). Receptor for activated protein kinase C1 regulates cell proliferation by modulating calcium signaling. Hypertension 58, 689–695. 10.1161/HYPERTENSIONAHA.111.174508 21844488 PMC3174333

[B27] Cheng D. Zhu X. Gillespie D. G. Jackson E. K. (2013). Role of RACK1 in the differential proliferative effects of neuropeptide Y(1-36) and peptide YY(1-36) in SHR vs. WKY preglomerular vascular smooth muscle cells. Am. J. Physiol. Renal Physiol. 304, F770–F780. 10.1152/ajprenal.00646.2012 23303411 PMC3602699

[B28] Chevalier R. L. Forbes M. S. Thornhill B. A. (2009). Ureteral obstruction as a model of renal interstitial fibrosis and obstructive nephropathy. Kidney Int. 75, 1145–1152. 10.1038/ki.2009.86 19340094

[B29] Cismowski M. J. Ma C. Ribas C. Xie X. Spruyt M. Lizano J. S.. (2000). Activation of heterotrimeric G-protein signaling by a ras-related protein. Implications for signal integration. J. Biol. Chem. 275, 23421–23424. 10.1074/jbc.C000322200 10840027

[B30] Cismowski M. J. Takesono A. Bernard M. L. Duzic E. Lanier S. M. (2001). Receptor-independent activators of heterotrimeric G-proteins. Life Sci. 68, 2301–2308. 10.1016/S0024-3205(01)01019-0 11358340

[B31] Cismowski M. J. Takesono A. Ma C. Lizano J. S. Xie X. Fuernkranz H.. (1999). Genetic screens in yeast to identify mammalian nonreceptor modulators of G-protein signaling. Nat. Biotechnol. 17, 878–883. 10.1038/12867 10471929

[B32] Cowley A. W. Jr. (1992). Long-term control of arterial blood pressure. Physiol. Rev. 72, 231–300. 1731371 10.1152/physrev.1992.72.1.231

[B33] Daaka Y. Pitcher J. A. Richardson M. Stoffel R. H. Robishaw J. D. Lefkowitz R. J. (1997). Receptor and G betagamma isoform-specific interactions with G protein-coupled receptor kinases. Proc. Natl. Acad. Sci. U.S.A. 94, 2180–2185. 10.1073/pnas.94.6.2180 9122168 PMC20061

[B34] Delmas P. Nomura H. Li X. Lakkis M. Luo Y. Segal Y.. (2002). Constitutive activation of G-proteins by polycystin-1 is antagonized by polycystin-2. J. Biol. Chem. 277, 11276–11283. 10.1074/jbc.M110483200 11786542

[B35] Felder R. A. Sanada H. Xu J. Yu P. Y. Wang Z. Watanabe H.. (2002). G protein-coupled receptor kinase 4 gene variants in human essential hypertension. Proc. Natl. Acad. Sci. U.S.A. 99, 3872–3877. 10.1073/pnas.062694599 11904438 PMC122616

[B36] Ferenbach D. A. Bonventre J. V. (2015). Mechanisms of maladaptive repair after AKI leading to accelerated kidney ageing and CKD. Nat. Rev. Nephrol. 11, 264–276. 10.1038/nrneph.2015.3 25643664 PMC4412815

[B37] Fischer E. Legue E. Doyen A. Nato F. Nicolas J. F. Torres V.. (2006). Defective planar cell polarity in polycystic kidney disease. Nat. Genet. 38, 21–23. 10.1038/ng1701 16341222

[B38] Furuya M. Nishiyama M. Kimura S. Suyama T. Naya Y. Ito H.. (2004). Expression of regulator of G protein signalling protein 5 (RGS5) in the tumour vasculature of human renal cell carcinoma. J. Pathol. 203, 551–558. 10.1002/path.1543 15095478

[B39] Gainetdinov R. R. Premont R. T. Caron M. G. Lefkowitz R. J. (2000). Reply: receptor specificity of G-protein-coupled receptor kinases. Trends Pharmacol. Sci. 21, 366–367. 10.1016/S0165-6147(00)01538-8 11050311

[B40] Garcia-Marcos M. Ghosh P. Farquhar M. G. (2015). GIV/Girdin transmits signals from multiple receptors by triggering trimeric G protein activation. J. Biol. Chem. 290, 6697–6704. 10.1074/jbc.R114.613414 25605737 PMC4358093

[B41] Ghosh P. Beas A. O. Bornheimer S. J. Garcia-Marcos M. Forry E. P. Johannson C. (2010). A G{alpha}i-GIV molecular complex binds epidermal growth factor receptor and determines whether cells migrate or proliferate. Mol. Biol. Cell 21, 2338–2354. 10.1091/mbc.E10-01-0028 20462955 PMC2893996

[B42] Gloerich M. Bos J. L. (2011). Regulating Rap small G-proteins in time and space. Trends Cell Biol. 21, 615–623. 10.1016/j.tcb.2011.07.001 21820312

[B43] Groves B. Abrahamsen H. Clingan H. Frantz M. Mavor L. Bailey J.. (2010). An inhibitory role of the G-protein regulator AGS3 in mTOR-dependent macroautophagy. PLoS ONE 5:e8877. 10.1371/journal.pone.0008877 20126274 PMC2811177

[B44] Guay-Woodford L. M. Wright C. J. Walz G. Churchill G. A. (2000). Quantitative trait loci modulate renal cystic disease severity in the mouse bpk model. J. Am. Soc. Nephrol. 11, 1253–1260. 10864581 10.1681/ASN.V1171253

[B45] Gurley S. B. Griffiths R. C. Mendelsohn M. E. Karas R. H. Coffman T. M. (2010). Renal actions of RGS2 control blood pressure. J. Am. Soc. Nephrol. 21, 1847–1851. 10.1681/ASN.2009121306 20847141 PMC3013999

[B46] Harrison E. M. Sharpe E. Bellamy C. O. McNally S. J. Devey L. Garden O. J.. (2008). Heat shock protein 90-binding agents protect renal cells from oxidative stress and reduce kidney ischemia-reperfusion injury. Am. J. Physiol. Renal Physiol. 295, F397–F405. 10.1152/ajprenal.00361.2007 18562631

[B47] Hercule H. C. Tank J. Plehm R. Wellner M. da Costa Goncalves A. C. Gollasch M.. (2007). Regulator of G protein signalling 2 ameliorates angiotensin II-induced hypertension in mice. Exp. Physiol. 92, 1014–1022. 10.1113/expphysiol.2007.038240 17644703

[B48] Hollinger S. Hepler J. R. (2002). Cellular regulation of RGS proteins: modulators and integrators of G protein signaling. Pharmacol. Rev. 54, 527–559. 10.1124/pr.54.3.527 12223533

[B49] Hong S. B. Oh H. Valera V. A. Baba M. Schmidt L. S. Linehan W. M. (2010). Inactivation of the FLCN tumor suppressor gene induces TFE3 transcriptional activity by increasing its nuclear localization. PLoS ONE 5:e15793. 10.1371/journal.pone.0015793 21209915 PMC3012117

[B50] Inanobe A. Takahashi K. Katada T. (1994). Association of the beta gamma subunits of trimeric GTP-binding proteins with 90-kDa heat shock protein, hsp90. J. Biochem. 115, 486–492. 8056761 10.1093/oxfordjournals.jbchem.a124363

[B51] Ingraffea J. Reczek D. Bretscher A. (2002). Distinct cell type-specific expression of scaffolding proteins EBP50 and E3KARP: EBP50 is generally expressed with ezrin in specific epithelia, whereas E3KARP is not. Eur. J. Cell Biol. 81, 61–68. 10.1078/0171-9335-00218 11893083

[B52] Insel P. A. Tang C. M. Hahntow I. Michel M. C. (2007). Impact of GPCRs in clinical medicine: monogenic diseases, genetic variants and drug targets. Biochim. Biophys. Acta 1768, 994–1005. 10.1016/j.bbamem.2006.09.029 17081496 PMC2169201

[B53] Jang H. S. Kim J. I. Noh M. Rhee M. H. Park K. M. (2014). Regulator of G protein signaling 2 (RGS2) deficiency accelerates the progression of kidney fibrosis. Biochim. Biophys. Acta 1842, 1733–1741. 10.1016/j.bbadis.2014.06.022 24973550

[B54] Jonasch E. Gao J. Rathmell W. K. (2014). Renal cell carcinoma. BMJ 349:g4797. 10.1136/bmj.g4797 25385470 PMC4707715

[B55] Kamal F. A. Travers J. G. Blaxall B. C. (2012). G protein-coupled receptor kinases in cardiovascular disease: why “where” matters. Trends Cardiovasc. Med. 22, 213–219. 10.1016/j.tcm.2012.07.023 23062971

[B56] Kemppainen R. J. Behrend E. N. (1998). Dexamethasone rapidly induces a novel ras superfamily member-related gene in AtT-20 cells. J. Biol. Chem. 273, 3129–3131. 10.1074/jbc.273.6.3129 9452419

[B57] Kemppainen R. J. Cox E. Behrend E. N. Brogan M. D. Ammons J. M. (2003). Identification of a glucocorticoid response element in the 3'-flanking region of the human Dexras1 gene. Biochim. Biophys. Acta 1627, 85–89. 10.1016/S0167-4781(03)00079-4 12818426

[B58] Kim E. Arnould T. Sellin L. Benzing T. Comella N. Kocher O.. (1999). Interaction between RGS7 and polycystin. Proc. Natl. Acad. Sci. U.S.A. 96, 6371–6376. 10.1073/pnas.96.11.6371 10339594 PMC26888

[B59] Kim H. R. Richardson J. van Eeden F. Ingham P. W. (2010). Gli2a protein localization reveals a role for Iguana/DZIP1 in primary ciliogenesis and a dependence of Hedgehog signal transduction on primary cilia in the zebrafish. BMC Biol. 8:65. 10.1186/1741-7007-8-65 20487519 PMC2890509

[B60] Kim W. J. Gersey Z. Daaka Y. (2012). Rap1GAP regulates renal cell carcinoma invasion. Cancer Lett. 320, 65–71. 10.1016/j.canlet.2012.01.022 22266190 PMC3319804

[B61] Knieke K. Hoff H. Maszyna F. Kolar P. Schrage A. Hamann A.. (2009). CD152 (CTLA-4) determines CD4 T cell migration *in vitro* and *in vivo*. PLoS ONE 4:e5702. 10.1371/journal.pone.0005702 19479036 PMC2682661

[B62] Konno D. Shioi G. Shitamukai A. Mori A. Kiyonari H. Miyata T.. (2008). Neuroepithelial progenitors undergo LGN-dependent planar divisions to maintain self-renewability during mammalian neurogenesis. Nat. Cell Biol. 10, 93–101. 10.1038/ncb1673 18084280

[B63] Koul H. Huh J. S. Rove K. O. Crompton L. Koul S. Meacham R. B.. (2011). Molecular aspects of renal cell carcinoma: a review. Am. J. Cancer Res. 1, 240–254. 21969126 PMC3180049

[B64] Kuiper R. P. Schepens M. Thijssen J. van Asseldonk M. van den Berg E. Bridge J.. (2003). Upregulation of the transcription factor TFEB in t(6;11)(p21;q13)-positive renal cell carcinomas due to promoter substitution. Hum. Mol. Genet. 12, 1661–1669. 10.1093/hmg/ddg178 12837690

[B65] Kwon M. Pavlov T. S. Nozu K. Rasmussen S. A. Ilatovskaya D. V. Lerch-Gaggl A.. (2012). G-protein signaling modulator 1 deficiency accelerates cystic disease in an orthologous mouse model of autosomal dominant polycystic kidney disease. Proc. Natl. Acad. Sci. U.S.A. 109, 21462–21467. 10.1073/pnas.1216830110 23236168 PMC3535663

[B66] Lenarczyk M. Pressly J. D. Arnett J. Regner K. R. Park F. (2015). Localization and expression profile of Group I and II Activators of G-protein Signaling in the kidney. J. Mol. Histol. 46, 123–136. 10.1007/s10735-014-9605-0 25533045 PMC4369180

[B67] Luijten M. N. Basten S. G. Claessens T. Vernooij M. Scott C. L. Janssen R.. (2013). Birt-Hogg-Dubé syndrome is a novel ciliopathy. Hum. Mol. Genet. 22, 4383–4397. 10.1093/hmg/ddt288 23784378 PMC3792695

[B68] Luyten A. Su X. Gondela S. Chen Y. Rompani S. Takakura A.. (2010). Aberrant regulation of planar cell polarity in polycystic kidney disease. J. Am. Soc. Nephrol. 21, 1521–1532. 10.1681/ASN.2010010127 20705705 PMC3013531

[B69] Ma B. Khazali A. Wells A. (2015). CXCR3 in Carcinoma Progression. Histol. Histopathol. 30, 781–792. 10.14670/HH-11-594 25663474 PMC4571436

[B70] Magalhaes A. C. Dunn H. Ferguson S. S. (2012). Regulation of GPCR activity, trafficking and localization by GPCR-interacting proteins. Br. J. Pharmacol. 165, 1717–1736. 10.1111/j.1476-5381.2011.01552.x 21699508 PMC3372825

[B71] Marwaha A. Lokhandwala M. F. (2006). Tempol reduces oxidative stress and restores renal dopamine D1-like receptor- G protein coupling and function in hyperglycemic rats. Am. J. Physiol. Renal Physiol. 291, F58–F66. 10.1152/ajprenal.00362.2005 16478977

[B72] Mathur M. Samuels H. H. (2007). Role of PSF-TFE3 oncoprotein in the development of papillary renal cell carcinomas. Oncogene 26, 277–283. 10.1038/sj.onc.1209783 16832349

[B73] Meloni A. M. Dobbs R. M. Pontes J. E. Sandberg A. A. (1993). Translocation (X;1) in papillary renal cell carcinoma. A new cytogenetic subtype. Cancer Genet. Cytogenet. 65, 1–6. 10.1016/0165-4608(93)90050-V 8431910

[B74] Men W. Martin T. A. Ruge F. Zhang N. Du P. Yang Y. Jiang W. G. (2015). Expression of claudins in human clear cell renal cell carcinoma. Cancer Genomics Proteomics 12, 1–8. 25560639

[B75] Meng X. M. Nikolic-Paterson D. J. Lan H. Y. (2014). Inflammatory processes in renal fibrosis. Nat. Rev. Nephrol. 10, 493–503. 10.1038/nrneph.2014.114 24981817

[B76] Miyamoto K. I. Itho M. (2001). Transcriptional regulation of the NPT2 gene by dietary phosphate. Kidney Int. 60, 412–415. 10.1046/j.1523-1755.2001.060002412.x 11473618

[B77] Miyazaki H. Anzai N. Ekaratanawong S. Sakata T. Shin H. J. Jutabha P.. (2005). Modulation of renal apical organic anion transporter 4 function by two PDZ domain-containing proteins. J. Am. Soc. Nephrol. 16, 3498–3506. 10.1681/ASN.2005030306 16236806

[B78] Morales F. C. Takahashi Y. Kreimann E. L. Georgescu M. M. (2004). Ezrin-radixin-moesin (ERM)-binding phosphoprotein 50 organizes ERM proteins at the apical membrane of polarized epithelia. Proc. Natl. Acad. Sci. U.S.A. 101, 17705–17710. 10.1073/pnas.0407974101 15591354 PMC539771

[B79] Morita K. Wakui H. Komatsuda A. Ohtani H. Miura A. B. Itoh H.. (1995). Induction of heat-shock proteins HSP73 and HSP90 in rat kidneys after ischemia. Ren. Fail. 17, 405–419. 10.3109/08860229509037605 7569112

[B80] Nadella R. Blumer J. B. Jia Kwon M. Akbulut T. Qian F.. (2010). Activator of G protein signaling 3 promotes epithelial cell proliferation in PKD. J. Am. Soc. Nephrol. 21, 1275–1280. 10.1681/ASN.2009121224 20488951 PMC2938587

[B81] Natochin M. Gasimov K. G. Artemyev N. O. (2001). Inhibition of GDP/GTP exchange on G alpha subunits by proteins containing G-protein regulatory motifs. Biochemistry 40, 5322–5328. 10.1021/bi015505w 11318657

[B82] Nishio S. Tian X. Gallagher A. R. Yu Z. Patel V. Igarashi P.. (2010). Loss of oriented cell division does not initiate cyst formation. J. Am. Soc. Nephrol. 21, 295–302. 10.1681/ASN.2009060603 19959710 PMC2834544

[B83] Padanilam B. J. Hammerman M. R. (1997). Ischemia-induced receptor for activated C kinase (RACK1) expression in rat kidneys. Am. J. Physiol. 272, F160–F166. 9124391 10.1152/ajprenal.1997.272.2.F160

[B84] Pang P. Jin X. Proctor B. M. Farley M. Roy N. Chin M. S.. (2015). RGS4 inhibits angiotensin II signaling and macrophage localization during renal reperfusion injury independent of vasospasm. Kidney Int. 87, 771–783. 10.1038/ki.2014.364 25469849 PMC4382433

[B85] Parnell S. C. Magenheimer B. S. Maser R. L. Rankin C. A. Smine A. Okamoto T.. (1998). The polycystic kidney disease-1 protein, polycystin-1, binds and activates heterotrimeric G-proteins *in vitro*. Biochem. Biophys. Res. Commun. 251, 625–631. 10.1006/bbrc.1998.9514 9792824

[B86] Parnell S. C. Magenheimer B. S. Maser R. L. Zien C. A. Frischauf A. M. Calvet J. P. (2002). Polycystin-1 activation of c-Jun N-terminal kinase and AP-1 is mediated by heterotrimeric G proteins. J. Biol. Chem. 277, 19566–19572. 10.1074/jbc.M201875200 11912216

[B87] Pattingre S. De Vries L. Bauvy C. Chantret I. Cluzeaud F. Ogier-Denis E.. (2003). The G-protein regulator AGS3 controls an early event during macroautophagy in human intestinal HT-29 cells. J. Biol. Chem. 278, 20995–21002. 10.1074/jbc.M300917200 12642577

[B88] Pattingre S. Petiot A. Codogno P. (2004). Analyses of Galpha-interacting protein and activator of G-protein-signaling-3 functions in macroautophagy. Methods Enzymol. 390, 17–31. 10.1016/S0076-6879(04)90002-X 15488168

[B89] Picascia A. Capobianco L. Iacovelli L. De Blasi A. (2004). Analysis of differential modulatory activities of GRK2 and GRK4 on Galphaq-coupled receptor signaling. Methods Enzymol. 390, 337–353. 10.1016/S0076-6879(04)90021-3 15488187

[B90] Potla U. Ni J. Vadaparampil J. Yang G. Leventhal J. S. Campbell K. N.. (2014). Podocyte-specific RAP1GAP expression contributes to focal segmental glomerulosclerosis-associated glomerular injury. J. Clin. Invest. 124, 1757–1769. 10.1172/JCI67846 24642466 PMC3973092

[B91] Prodromou N. V. Thompson C. L. Osborn D. P. Cogger K. F. Ashworth R. Knight M. M.. (2012). Heat shock induces rapid resorption of primary cilia. J. Cell Sci. 125, 4297–4305. 10.1242/jcs.100545 22718348 PMC3516438

[B92] Ramírez V. Mejíia-Vilet J. M. Hernández D. Gamba G. Bobadilla N. A. (2008). Radicicol, a heat shock protein 90 inhibitor, reduces glomerular filtration rate. Am. J. Physiol. Renal Physiol. 295, F1044–F1051. 10.1152/ajprenal.90278.2008 18667483

[B93] Reczek D. Berryman M. Bretscher A. (1997). Identification of EBP50: a PDZ-containing phosphoprotein that associates with members of the ezrin-radixin-moesin family. J. Cell Biol. 139, 169–179. 10.1083/jcb.139.1.169 9314537 PMC2139813

[B94] Regner K. R. Nozu K. Lanier S. M. Blumer J. B. Avner E. D. Sweeney W. E. Jr. Park F. (2011). Loss of activator of G-protein signaling 3 impairs renal tubular regeneration following acute kidney injury in rodents. FASEB J. 25, 1844–1855. 10.1096/fj.10-169797 21343176 PMC3101034

[B95] Ritter S. L. Hall R. A. (2009). Fine-tuning of GPCR activity by receptor-interacting proteins. Nat. Rev. Mol. Cell Biol. 10, 819–830. 10.1038/nrm2803 19935667 PMC2825052

[B96] Rochdi M. D. Watier V. La Madeleine C. Nakata H. Kozasa T. Parent J. L. (2002). Regulation of GTP-binding protein alpha q (Galpha q) signaling by the ezrin-radixin-moesin-binding phosphoprotein-50 (EBP50). J. Biol. Chem. 277, 40751–40759. 10.1074/jbc.M207910200 12193606

[B97] Ron D. Chen C. H. Caldwell J. Jamieson L. Orr E. Mochly-Rosen D. (1994). Cloning of an intracellular receptor for protein kinase C: a homolog of the beta subunit of G proteins. Proc. Natl. Acad. Sci. U.S.A. 91, 839–843. 10.1073/pnas.91.3.839 8302854 PMC521407

[B98] Roy A. A. Baragli A. Bernstein L. S. Hepler J. R. Hébert T. E. Chidiac P. (2006). RGS2 interacts with Gs and adenylyl cyclase in living cells. Cell. Signal. 18, 336–348. 10.1016/j.cellsig.2005.05.004 16095880

[B99] Roy A. A. Lemberg K. E. Chidiac P. (2003). Recruitment of RGS2 and RGS4 to the plasma membrane by G proteins and receptors reflects functional interactions. Mol. Pharmacol. 64, 587–593. 10.1124/mol.64.3.587 12920194

[B100] Roychowdhury S. Rasenick M. M. (1994). Tubulin-G protein association stabilizes GTP binding and activates GTPase: cytoskeletal participation in neuronal signal transduction. Biochemistry 33, 9800–9805. 10.1021/bi00198a052 8068660

[B101] Roychowdhury S. Panda D. Wilson L. Rasenick M. M. (1999). G protein alpha subunits activate tubulin GTPase and modulate microtubule polymerization dynamics. J. Biol. Chem. 274, 13485–13490. 10.1074/jbc.274.19.13485 10224115

[B102] Rubinfeld B. Munemitsu S. Clark R. Conroy L. Watt K. Crosier W. J.. (1991). Molecular cloning of a GTPase activating protein specific for the Krev-1 protein p21rap1. Cell 65, 1033–1042. 10.1016/0092-8674(91)90555-D 1904317

[B103] Rusai K. Prokai A. Juanxing C. Meszaros K. Szalay B. Pásti K.. (2013). Dexamethasone protects from renal ischemia/reperfusion injury: a possible association with SGK-1. Acta Physiol. Hung. 100, 173–185. 10.1556/APhysiol.100.2013.001 23524182

[B104] Saburi S. Hester I. Fischer E. Pontoglio M. Eremina V. Gessler M.. (2008). Loss of Fat4 disrupts PCP signaling and oriented cell division and leads to cystic kidney disease. Nat. Genet. 40, 1010–1015. 10.1038/ng.179 18604206

[B105] Sato M. Hiraoka M. Suzuki H. Bai Y. Kurotani R. Yokoyama U.. (2011). Identification of transcription factor E3 (TFE3) as a receptor-independent activator of Galpha16: gene regulation by nuclear Galpha subunit and its activator. J. Biol. Chem. 286, 17766–17776. 10.1074/jbc.M111.219816 21454667 PMC3093852

[B106] Sato P. Y. Chuprun J. K. Schwartz M. Koch W. J. (2015). The evolving impact of g protein-coupled receptor kinases in cardiac health and disease. Physiol. Rev. 95, 377–404. 10.1152/physrev.00015.2014 25834229 PMC4551214

[B107] Satoh K. Wakui H. Komatsuda A. Nakamoto Y. Miura A. B. Itoh H.. (1994). Induction and altered localization of 90-kDa heat-shock protein in rat kidneys with cisplatin-induced acute renal failure. Ren. Fail. 16, 313–323. 10.3109/08860229409044872 8059015

[B108] Schappi J. M. Krbanjevic A. Rasenick M. M. (2014). Tubulin, actin and heterotrimeric G proteins: coordination of signaling and structure. Biochim. Biophys. Acta 1838, 674–681. 10.1016/j.bbamem.2013.08.026 24071592 PMC3877202

[B109] Seeger-Nukpezah T. Proia D. A. Egleston B. L. Nikonova A. S. Kent T. Cai K. Q.. (2013). Inhibiting the HSP90 chaperone slows cyst growth in a mouse model of autosomal dominant polycystic kidney disease. Proc. Natl. Acad. Sci. U.S.A. 110, 12786–12791. 10.1073/pnas.1301904110 23858461 PMC3732984

[B110] Siderovski D. P. Willard F. S. (2005). The GAPs, GEFs, and GDIs of heterotrimeric G-protein alpha subunits. Int. J. Biol. Sci. 1, 51–66. 10.7150/ijbs.1.51 15951850 PMC1142213

[B111] Siedlecki A. M. Jin X. Thomas W. Hruska K. A. Muslin A. J. (2011). RGS4, a GTPase activator, improves renal function in ischemia-reperfusion injury. Kidney Int. 80, 263–271. 10.1038/ki.2011.63 21412219 PMC3221244

[B112] Silini A. Ghilardi C. Figini S. Sangalli F. Fruscio R. Dahse R. (2012). Regulator of G-protein signaling 5 (RGS5) protein: a novel marker of cancer vasculature elicited and sustained by the tumor's proangiogenic microenvironment. Cell. Mol. Life Sci. 69, 1167–1178. 10.1007/s00018-011-0862-8 22130514 PMC3299962

[B113] Smithline Z. B. Nikonova A. S. Hensley H. H. Cai K. Q. Egleston B. L. Proia D. A.. (2014). Inhibiting heat shock protein 90 (HSP90) limits the formation of liver cysts induced by conditional deletion of Pkd1 in mice. PLoS ONE 9:e114403. 10.1371/journal.pone.0114403 25474361 PMC4256400

[B114] Snow B. E. Krumins A. M. Brothers G. M. Lee S. F. Wall M. A. Chung S.. (1998). A G protein gamma subunit-like domain shared between RGS11 and other RGS proteins specifies binding to Gbeta5 subunits. Proc. Natl. Acad. Sci. U.S.A. 95, 13307–13312. 10.1073/pnas.95.22.13307 9789084 PMC23793

[B115] Spilker C. Kreutz M. R. (2010). RapGAPs in brain: multipurpose players in neuronal Rap signalling. Eur. J. Neurosci. 32, 1–9. 10.1111/j.1460-9568.2010.07273.x 20576033

[B116] Takesono A. Cismowski M. J. Ribas C. Bernard M. Chung P. Hazard S. III. (1999). Receptor-independent activators of heterotrimeric G-protein signaling pathways. J. Biol. Chem. 274, 33202–33205. 10.1074/jbc.274.47.33202 10559191

[B117] Tobin A. B. (2008). G-protein-coupled receptor phosphorylation: where, when and by whom. Br. J. Pharmacol. 153(Suppl. 1), S167–S176. 10.1038/sj.bjp.0707662 18193069 PMC2268057

[B118] Trivedi M. Lokhandwala M. F. (2005). Rosiglitazone restores renal D1A receptor-Gs protein coupling by reducing receptor hyperphosphorylation in obese rats. Am. J. Physiol. Renal Physiol. 289, F298–F304. 10.1152/ajprenal.00362.2004 15798088

[B119] Tsygankova O. M. Prendergast G. V. Puttaswamy K. Wang Y. Feldman M. D. Wang H.. (2007). Downregulation of Rap1GAP contributes to Ras transformation. Mol. Cell. Biol. 27, 6647–6658. 10.1128/MCB.00155-07 17646383 PMC2099240

[B120] Tu Y. Wu C. (1999). Cloning, expression and characterization of a novel human Ras-related protein that is regulated by glucocorticoid hormone. Biochim. Biophys. Acta 1489, 452–456. 10.1016/S0167-4781(99)00197-9 10673050

[B121] Vaiskunaite R. Adarichev V. Furthmayr H. Kozasa T. Gudkov A. Voyno-Yasenetskaya T. A. (2000). Conformational activation of radixin by G13 protein alpha subunit. J. Biol. Chem. 275, 26206–26212. 10.1074/jbc.M001863200 10816569

[B122] Vaiskunaite R. Kozasa T. Voyno-Yasenetskaya T. A. (2001). Interaction between the G alpha subunit of heterotrimeric G(12) protein and Hsp90 is required for G alpha(12) signaling. J. Biol. Chem. 276, 46088–46093. 10.1074/jbc.M108711200 11598136

[B123] Virman J. Soini Y. Kujala P. Luukkaala T. Salminen T. Sunela K.. (2014). Claudins as prognostic factors for renal cell cancer. Anticancer Res. 34, 4181–4187. 25075044

[B124] Volta V. Beugnet A. Gallo S. Magri L. Brina D. Pesce E.. (2013). RACK1 depletion in a mouse model causes lethality, pigmentation deficits and reduction in protein synthesis efficiency. Cell. Mol. Life Sci. 70, 1439–1450. 10.1007/s00018-012-1215-y 23212600 PMC11113757

[B125] Walker C. (1998). Molecular genetics of renal carcinogenesis. Toxicol. Pathol. 26, 113–120. 10.1177/019262339802600113 9502393

[B126] Wang H. Misaki T. Taupin V. Eguchi A. Ghosh P. Farquhar M. G. (2015). GIV/girdin links vascular endothelial growth factor signaling to AKT survival signaling in podocytes independent of nephrin. J. Am. Soc. Nephrol. 26, 314–327. 10.1681/ASN.2013090985 25012178 PMC4310647

[B127] Weinman E. J. Steplock D. Wang Y. Shenolikar S. (1995). Characterization of a protein cofactor that mediates protein kinase A regulation of the renal brush border membrane Na(+)-H+ exchanger. J. Clin. Invest. 95, 2143–2149. 10.1172/JCI117903 7738182 PMC295815

[B128] Weterman M. A. van Groningen J. J. den Hartog A. Geurts van Kessel A. (2001). Transformation capacities of the papillary renal cell carcinoma-associated PRCCTFE3 and TFE3PRCC fusion genes. Oncogene 20, 1414–1424. 10.1038/sj.onc.1204213 11313885

[B129] Weterman M. A. Wilbrink M. Geurts van Kessel A. (1996a). Fusion of the transcription factor TFE3 gene to a novel gene, PRCC, in t(X;1)(p11;q21)-positive papillary renal cell carcinomas. Proc. Natl. Acad. Sci. U.S.A. 93, 15294–15298. 10.1073/pnas.93.26.15294 8986805 PMC26398

[B130] Weterman M. A. Wilbrink M. Janssen I. Janssen H. A. van den Berg E. Fisher S. E.. (1996b). Molecular cloning of the papillary renal cell carcinoma-associated translocation (X;1)(p11;q21) breakpoint. Cytogenet. Cell Genet. 75, 2–6. 10.1159/000134444 8995477

[B131] Weterman M. J. van Groningen J. J. Jansen A. van Kessel A. G. (2000). Nuclear localization and transactivating capacities of the papillary renal cell carcinoma-associated TFE3 and PRCC (fusion) proteins. Oncogene 19, 69–74. 10.1038/sj.onc.1203255 10644981

[B132] Wettschureck N. Offermanns S. (2005). Mammalian G proteins and their cell type specific functions. Physiol. Rev. 85, 1159–1204. 10.1152/physrev.00003.2005 16183910

[B133] Whitesell L. Lin N. U. (2012). HSP90 as a platform for the assembly of more effective cancer chemotherapy. Biochim. Biophys. Acta 1823, 756–766. 10.1016/j.bbamcr.2011.12.006 22222203

[B134] Woo D. D. Tabancay A. P. Jr. Wang C. J. (1997). Microtubule active taxanes inhibit polycystic kidney disease progression in cpk mice. Kidney Int. 51, 1613–1618. 10.1038/ki.1997.222 9150481

[B135] Xiao Z. Wan Q. Du Q. Zheng Z. (2012). Galpha/LGN-mediated asymmetric spindle positioning does not lead to unequal cleavage of the mother cell in 3-D cultured MDCK cells. Biochem. Biophys. Res. Commun. 420, 888–894. 10.1016/j.bbrc.2012.03.095 22469469 PMC3334408

[B136] Yan K. Greene E. Belga F. Rasenick M. M. (1996). Synaptic membrane G proteins are complexed with tubulin *in situ*. J. Neurochem. 66, 1489–1495. 10.1046/j.1471-4159.1996.66041489.x 8627303

[B137] Yang S. Li Y. P. Liu T. He X. Yuan X. Li C.. (2013). Mx1-cre mediated Rgs12 conditional knockout mice exhibit increased bone mass phenotype. Genesis 51, 201–209. 10.1002/dvg.22373 23349096 PMC3908791

[B138] Yeh C. Li A. Chuang J. Z. Saito M. Cáceres A. Sung C. H. (2013). IGF-1 activates a cilium-localized noncanonical Gbetagamma signaling pathway that regulates cell-cycle progression. Dev. Cell 26, 358–368. 10.1016/j.devcel.2013.07.014 23954591 PMC3790638

[B139] Yu W. Kong T. Beaudry S. Tran M. Negoro H. Yanamadala V.. (2010). Polycystin-1 protein level determines activity of the Galpha12/JNK apoptosis pathway. J. Biol. Chem. 285, 10243–10251. 10.1074/jbc.M109.070821 20106977 PMC2856229

[B140] Yu W. Ritchie B. J. Su X. Zhou J. Meigs T. E. Denker B. M. (2011). Identification of polycystin-1 and Galpha12 binding regions necessary for regulation of apoptosis. Cell. Signal. 23, 213–221. 10.1016/j.cellsig.2010.09.005 20837139 PMC2998059

[B141] Yuan X. Cao J. Liu T. Li Y. P. Scannapieco F. He X.. (2015). Regulators of G protein signaling 12 promotes osteoclastogenesis in bone remodeling and pathological bone loss. Cell Death Differ. [Epub ahead of print]. 10.1038/cdd.2015.45 25909889 PMC4816106

[B142] Zhang J. Yao Y. Xiao F. Lan X. Yu C. Zhang Y.. (2013). Administration of dexamethasone protects mice against ischemia/reperfusion induced renal injury by suppressing PI3K/AKT signaling. Int. J. Clin. Exp. Pathol. 6, 2366–2375. 24228098 PMC3816805

[B143] Zheng S. Yu P. Zeng C. Wang Z. Yang Z. Andrews P. M.. (2003). Galpha12- and Galpha13-protein subunit linkage of D5 dopamine receptors in the nephron. Hypertension 41, 604–610. 10.1161/01.HYP.0000057422.75590.D7 12623966

[B144] Zheng Z. Zhu H. Wan Q. Liu J. Xiao Z. Siderovski D. P.. (2010). LGN regulates mitotic spindle orientation during epithelial morphogenesis. J. Cell Biol. 189, 275–288. 10.1083/jcb.200910021 20385777 PMC2856901

[B145] Zuber A. M. Singer D. Penninger J. M. Rossier B. C. Firsov D. (2007). Increased renal responsiveness to vasopressin and enhanced V2 receptor signaling in RGS2-/- mice. J. Am. Soc. Nephrol. 18, 1672–1678. 10.1681/ASN.2007010032 17475820

